# Adjustment for time-invariant and time-varying confounders in ‘unexplained residuals’ models for longitudinal data within a causal framework and associated challenges

**DOI:** 10.1177/0962280218756158

**Published:** 2018-02-16

**Authors:** KF Arnold, GTH Ellison, SC Gadd, J Textor, PWG Tennant, A Heppenstall, MS Gilthorpe

**Affiliations:** 1 Leeds Institute for Data Analytics, University of Leeds, Leeds, UK; 2 School of Medicine, University of Leeds, Leeds, UK; 3 Tumor Immunology Lab, Radboud University Medical Centre, Nijmegen, The Netherlands; 4 School of Healthcare, University of Leeds, Leeds, UK; 5 School of Geography, University of Leeds, Leeds, UK

**Keywords:** Unexplained residuals model, conditional regression model, conditional analysis, conditional growth, conditional weight, conditional size, directed acyclic graph, causal inference, lifecourse epidemiology

## Abstract

‘Unexplained residuals’ models have been used within lifecourse epidemiology to model an exposure measured longitudinally at several time points in relation to a distal outcome. It has been claimed that these models have several advantages, including: the ability to estimate multiple total causal effects in a single model, and additional insight into the effect on the outcome of greater-than-expected increases in the exposure compared to traditional regression methods. We evaluate these properties and prove mathematically how adjustment for confounding variables must be made within this modelling framework. Importantly, we explicitly place unexplained residual models in a causal framework using directed acyclic graphs. This allows for theoretical justification of appropriate confounder adjustment and provides a framework for extending our results to more complex scenarios than those examined in this paper. We also discuss several interpretational issues relating to unexplained residual models within a causal framework. We argue that unexplained residual models offer no additional insights compared to traditional regression methods, and, in fact, are more challenging to implement; moreover, they artificially reduce estimated standard errors. Consequently, we conclude that unexplained residual models, if used, must be implemented with great care.

## 1 Background

**Figure 1.**
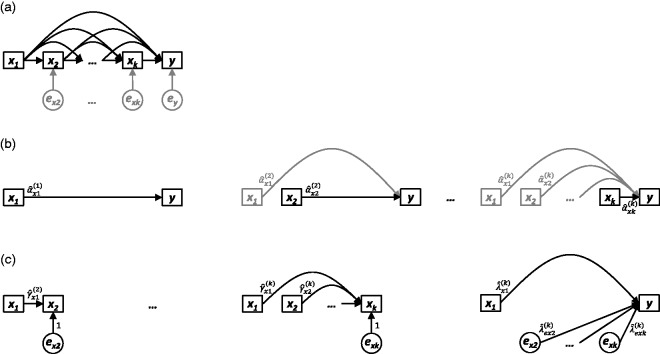
(a) Nonparametric causal diagram (DAG) representing the hypothesised data-generating process for *k* longitudinal measurements of exposure *x* (i.e. *x_1_*,*x_2_* ,…, *x_k_*) and one distal outcome *y* . The terms *e_x2_*,…,*e_xk_* and *e_y_* represent all unexplained causes of *x_2_*,…,*x_k_* and *y* , respectively, and are included to explicitly reflect uncertainty in all endogenous nodes (whether modelled or not). (b) Path diagrams depicting the *k* standard regression models that would be constructed to estimate the total causal effect of each of *x*_1_,*x*_2_,…,*x_k_* on *y* (i.e. equation (5)). For each model, only the final coefficient may be interpreted as a total causal effect; all other coefficients are greyed to illustrate that no such interpretation should be made for them. (c) Path diagrams depicting the UR model, consisting of *k*  − 1 preparation regressions (i.e. equation (6)) and a final composite regression model (i.e. equation (7), with *i*  = *k* ).

Within the field of lifecourse epidemiology, there is substantial interest in modelling the relationship between an exposure *x* measured longitudinally at several time points (i.e. $x_{1},x_{2},\ldots ,x_{k}$) and a subsequent outcome *y* measured once later in life (hereafter referred to as a distal outcome); such a relationship can be helpfully summarised in Figure 1(a) in the form of a directed acyclic graph (DAG).^1^ DAGs are pictorial representations of hypothesised causal relationships between variables in which: variables (nodes) are connected via unidirectional arrows (directed edges), which represent direct causal relationships; and no directed loops (i.e. circular paths) between variables are permitted. Nodes may be either: endogenous, having at least one causally preceding variable represented in the graph; or exogenous, having none.^2^ All unexplained causes of the endogenous nodes $x_{2},\ldots ,x_{k},y$ in Figure 1(a) are represented by the variables $e_{x2},\ldots ,e_{\mathrm{xk}},e_{y}$, respectively. While there are many useful applications for DAGs in epidemiologic research, perhaps the most beneficial is their ability to identify suitable sets of covariates for removing bias due to confounding between an exposure and outcome,^3,4^ which occurs whenever both variables share one or more common causes. For this reason, DAGs are increasingly being used in epidemiology, as they provide a framework for estimating the total causal effect of an exposure on an outcome.^4^

Using a causal framework to (correctly) model the scenario in Figure 1(a) may also have additional utility in identifying and quantifying important periods of change in the exposure that are causally related to the outcome. However, one challenge to such applications is that successive measurements of an exposure over time may be highly correlated with one another and therefore likely to suffer collinearity when analysed in relation to a distal outcome. Consequently, there has been extensive debate regarding the best way to model these types of longitudinally measured variables; a recent review^5^ of analytical and modelling techniques has identified a range of different approaches, including z-score plots, regression with change scores, multilevel and latent growth curve models, and growth mixture models. Nonetheless, one of the most straightforward methods in use is a series of standard multivariable regression models.

### 1.1 Standard regression method

When using this approach, each longitudinal measurement of the exposure variable is treated as a separate entity that is subject to confounding by all previous measurements of that variable – the total number of models needed therefore being equal to the total number of time points at which the exposure has been measured.

As an example, the simplest scenario would involve just two measurements of the exposure *x* (i.e. *x*_1_ and *x*_2_, measured at time points 1 and 2, respectively), and a distal outcome, *y*, where all variables are continuous in nature. Here, two standard regression models (denoted y^S(i), for i=1,2) would need to be constructed to estimate the total causal effect of each distinct measurement of *x* on *y*, i.e.

(1) y^S(1)=α^0(1)+α^x1(1)x1

(2) y^S(2)=α^0(2)+α^x1(2)x1+α^x2(2)x2.

Importantly, to estimate the total causal effect of *x*_1_ on *y* in equation (1), adjustment for *x*_2_ is inappropriate, as it lies on the causal path between *x*_1_ and *y* (i.e. *x*_2_ is a mediator); in fact, adjustment for *x*_2_ might invoke bias in the causal interpretation due to a phenomenon known as the ‘reversal paradox’.^5–7^ In contrast, to estimate the total causal effect of *x*_2_ on *y* in equation (2), adjustment for *x*_1_ *is* appropriate, since it confounds the desired relationship (i.e. *x*_1_ causally precedes both *x*_2_ and *y*, potentially creating a spurious relationship between them). For this reason, in either model, it is *only* possible to interpret the coefficient of the last/most recent measurement of *x* (the exposure) as a total causal effect,^1^ which encompasses all direct and indirect causal pathways between an exposure and outcome. No such interpretation is possible (nor should it be attempted) for the coefficient of the earlier measurement of *x* in equation (2), as it operates purely as a confounder.

### 1.2 Unexplained residuals method

To circumvent the need for multiple models, Keijzer-Veen^8^ has suggested an alternative approach that would combine the information contained within each of the two separate models (equations (1) and (2)) into a single composite regression model using ‘unexplained residuals’. As originally proposed,^9^ such a model allows the researcher to quantify the total effects of both the initial measurement of *x* (i.e. $x_{1})$ and subsequent change in *x* on the outcome *y*. The proposed approach contains two steps but is straightforward in principle.

First, the most recent measurement of *x* (i.e. *x*_2_) is regressed on the earlier measurement of *x* (i.e. *x*_1_):

(3) x2=γ^0(2)+γ^x1(2)x1+ex2.

This produces a measure of each observation’s ‘expected’ value of *x*_2_ as predicted by its value of *x*_1_. The difference between the expected value of *x*_2_ (i.e. γ^0(2)+γ^x1(2)x1) and the observed value of *x*_2_ amounts to the residual term $e_{x2}$. Put another way, $e_{x2}$ represents the part of *x*_2_ ‘unexplained’ by *x*_1_.

Second, *y* is regressed on both the initial exposure *x*_1_ and subsequent residual term $e_{x2}:$

(4) y^UR(2)=λ^0(2)+λ^x1(2)x1+λ^ex2(2)ex2.

According to Keijzer-Veen et al.,^9^ the key advantages of conducting regression using the composite ‘unexplained residuals’ (UR) model (4) are that:

1. The UR model produces the same estimated outcome values as the standard regression model in equation (2) (i.e. y^S(2)=y^UR(2));
2. The estimated total effect sizes (coefficient values) produced by individual standard regression models (equations (1) and (2)) are equal to those estimated within the UR model (i.e. α^x1(1)=λ^x1(2) and α^x2(2)=λ^ex2(2)); thus, multiple coefficients in a single model may be interpreted;
3. The UR model provides additional insight (via the coefficient λ^ex2(2) in equation (4)) into the effect of *x* increasing *more than expected* upon *y*; and
4. The initial exposure *x*_1_ and subsequent residual term $e_{x2}$ are mathematically independent (i.e. orthogonal).

Succinctly, the two models y^S(2) and y^UR(2) are algebraically equivalent, but y^UR(2) makes interpretation of the separate influence of the initial measurement of the exposure *x* (i.e. *x*_1_) *and* subsequent changes in *x* more straightforward than do (multiple) standard regression models y^S(1) and y^S(2).

Within the epidemiological literature, UR models have been used under a number of different names. In addition to ‘regression with unexplained residuals’ (as first proposed by Keijzer-Veen et al.^9–11^), other studies have referred to: ‘unexplained residual regression’^12^; ‘method of unexplained residuals’^13^; ‘conditional linear regression’^12^; ‘conditional (regression) models’^5,14^; ‘regression with conditional growth measures’^14^; ‘conditional growth models’^15–18^; ‘conditional weight models’^19^; and ‘conditional (regression) analysis’.^20–24^ The terms ‘conditional growth’ and ‘conditional size’ – and additional variations thereof – are also commonly used to refer to the difference between observed and expected size measurements.^5,15,18,25–39^ To avoid further confusion, the residual term representing the difference between the observed and expected values of an exposure produced in the manner proposed by Keijzer-Veen et al. (as in equation (3)) will be henceforth referred to as the ‘*unexplained residuals (UR) term*’, and the models themselves (as in equation (4)) will be referred to as ‘*unexplained residuals (UR) models*’.

Despite the numerous names given to these models, the process remains essentially the same as that first proposed. Indeed, several authors have extended the original model to examine scenarios involving several measurements of an exposure *x* (i.e. $x_{1},x_{2},\ldots ,x_{k}$); UR models in these extended applications thus include several UR terms.^5,12,13,16–41^ In general, each UR term $e_{\mathrm{xi}}$ is derived from the regression of each measured value *x_i_* on all previous measurements $x_{1},x_{2},\ldots ,x_{i-1}$, for 2≤i≤k,^12,16,18–22,24,25,27,29,31–34,36,39,40^ though some researchers have deviated from this procedure^13,26,35,37,41^; the outcome *y* is then regressed on *x*_1_ and all subsequent UR terms $e_{x2},e_{x3},\ldots ,e_{\mathrm{xk}}$.

Many researchers have further extended the original UR models by adjusting for additional confounding variables (i.e. over and above the confounding of prior measurements of the exposure), though there is, as yet, little consensus as to whether or how such adjustments should be performed. For example, Horta et al.^16^ made no adjustments for potential confounders when deriving their UR terms, but did make adjustments within their composite UR model. In contrast, Gandhi et al.^18^ adjusted for just one potential confounder (gender) when creating their UR terms, but also made further adjustments to the composite UR model (for gender and other variables). Adair et al.^25^ created their UR terms using site- and sex-stratified linear regressions that were also adjusted for age, and made further adjustments for age, sex, and study site in their subsequent composite UR models. Indeed, there are many other examples of different approaches to confounder adjustment, but none of these have been adequately and explicitly justified by the researchers concerned, even though it appears that they did so in order to make causal inferences.

## 2 Research aims

The potential impact of using alternative approaches to adjust for confounding when constructing and using UR terms has yet to be fully evaluated. Indeed, Keijzer-Veen et al.^9^ did not address confounding variables in their original paper, and there has been little to no discussion or analysis of this issue by subsequent authors using this approach. It therefore remains unclear whether UR models that include confounders offer the same purported benefits as those lacking (or ignoring) confounders, and there is no clear indication of how potential confounders should be treated by analyses using these models. This is an issue of particular relevance to researchers seeking to infer causality from individual coefficient estimates, since inappropriate adjustment for covariates (which includes both the failure to adjust for genuine confounders and the adjustment for mediators mistaken for confounders) can lead to biased causal inferences. For this reason, UR models are likely to have limited practical utility unless they are able to accommodate confounding variables appropriately. The fact that UR models have not been developed or analysed within a causal framework also creates uncertainty about their utility for making causal inferences.

Therefore, the aims of the present study were to: (1) confirm that the approach proposed by Keijzer-Veen et al. may be extended to a scenario involving *k* longitudinal measurements of an exposure *x* in the absence of any additional confounding; (2) determine whether it is possible (and if so, how might it be possible) to adjust for additional confounders within the UR modelling framework; (3) evaluate the benefits of UR models claimed by Keijzer-Veen et al.; and (4) offer recommendations for future use of UR models The present study examines two very different types of potential confounders: time-invariant (which require/provide measurements taken at a single time point and remain constant across the lifecourse, e.g. sex); and time-varying (for which measurements are collected at multiple time points across the lifecourse – usually concurrent to measurements of the exposure – because the value of the variable may change, e.g. socioeconomic position).

**Figure 2.**
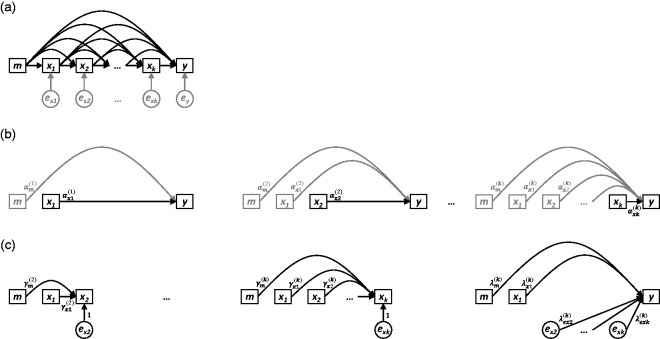
(a) Nonparametric causal diagram (DAG) representing the hypothesised data-generating process for *k* longitudinal measurements of exposure *x* (i.e. *x*_1_,*x*_2_,…,*x_k_*), one distal outcome *y* , and one time-invariant confounder *m* . The terms *e_m_*, *e_x_*_1_,…,*e_xk_* and *e_y_* represent all unexplained causes of *m*, *x*_1_,…,*x_k_*, and *y*, respectively, and are included to explicitly reflect uncertainty in all endogenous nodes (whether modelled or not).(b) Path diagrams depicting the *k* standard regression models that would be constructed to estimate the total causal effect of each of *x*_1_,*x*_2_,…,*x_k_* on *y* (i.e. equation (9)). For each model, only the final coefficient may be interpreted as a total causal effect; all other coefficients are greyed to illustrate that no such interpretation should be made for them. (c) Path diagrams depicting the UR model, consisting of *k*  − 1 preparation regressions (i.e. equation (10)) and a final composite regression model (i.e. equation (11), with *i*  = *k* ).

**Figure 3.**
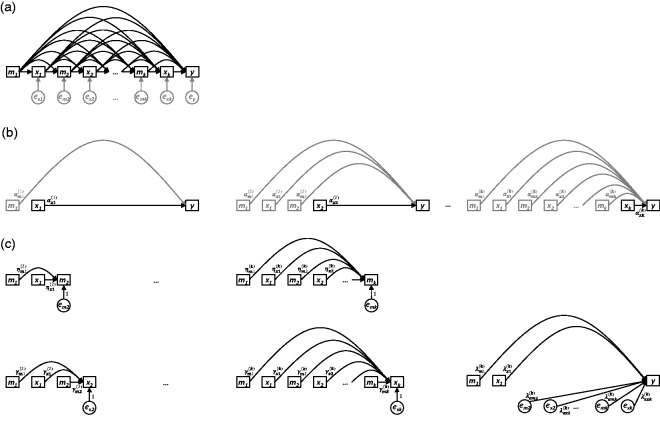
(a) Nonparametric causal diagram (DAG) representing the hypothesised data-generating process for *k* longitudinal measurements of exposure *x* (i.e. *x*_1_,*x*_2_,…,*x_k_* ), one distal outcome *y*, and *k* longitudinal measurements of one time-varying confounder *m*_1_,*m*_2_,…,*m*_*k*_ . The terms *e*_*m*2_, …, *e_mk_*, *e_x1_*,…,*e_xk_* and *e_y_* represent all unexplained causes of *m_2_*,…, *m_k_*, *x_1_* ,…, *x_k_*, and *y*, respectively, and are included to explicitly reflect uncertainty in all endogenous nodes (whether modelled or not). (b) Path diagrams depicting the *k* standard regression models that would be constructed to estimate the total causal effect of each of *x_1_*, *x_2_* ,…, *x_k_* on *y* (i.e. equation (12)). For each model, only the final coefficient may be interpreted as a total causal effect; all other coefficients are greyed to illustrate that no such interpretation should be made for them. (c) Path diagrams depicting the UR model, consisting of 2(*k*  − 1) preparation regressions (i.e. equations (13) and (14)) and a final composite regression model (i.e. equation (15), with *i*  = *k* ).

These aims are summarised in the DAGs presented in Figures 1(a), 2(a), and 3(a), which depict three general scenarios drawn from lifecourse epidemiology, each of which will be examined in the analyses that follow. Each DAG relates *k* longitudinally measured exposure variables $x_{1},x_{2},\ldots ,x_{k}$ (i.e. *x* measured at time points 1,2,…,k) to a distal outcome *y* (measured at some point either concurrent to or following *k*) under three very different circumstances: (1a) in the absence of any additional confounders; (2a) in the presence of an additional time-invariant confounder *m*; and (3a) in the presence of an additional time-varying confounder $m_{1},m_{2},\ldots ,m_{k}$. All DAGs are drawn forwardly saturated (i.e. where each node may causally affect all future nodes), and all unexplained causes of endogenous nodes are represented by the variable *e* and depicted as independent (i.e. we assume no unobserved confounding). The explicit inclusion of these three DAGs in Figures 1(a), 2(a), and 3(a) is intended not only to visually illustrate each of the scenarios that will be examined, but also, importantly, to situate the analyses that follow within a causal framework.

Sections 3 through 9, which follow, provide: the three key properties of UR models that will be evaluated for the scenarios in Figures 1(a), 2(a), and 3(a) (§3); DAG-based and mathematical examinations of the UR models for the scenarios given in Figure 1(a) (§4), 2(a) (§5), and 3(a) (§6); a discussion of several interpretational issues that arise for UR models when placed within a causal framework, including an evaluation of the claim that UR models provide greater insight than standard regression methods (§7); an argument outlining how UR models produce artificially reduced standard errors (SEs) and how this might be corrected (§8); and recommendations for future use and interpretation of UR models, particularly as these relate to the inclusion of confounders (§9).

## 3 Key properties of UR models

In the following sections, we evaluate the mathematical properties of the original UR models after extending them to include *k* measurements of a continuous exposure *x*: in the absence of any additional confounding (§4); in the presence of a single additional time-invariant confounder *m* (§5); and in the presence of a single additional time-varying confounder with sequential values $m_{1},m_{2},\ldots ,m_{k}$ (§6). These properties are:

- **Property (i):** The outcome values predicted by the final standard regression model (for the final measurement of the exposure variable, *x_k_*) are equal to those predicted by the composite UR model.
- **Property (ii):** The estimated coefficient for *x*_1_ in the initial standard regression model (for the first measurement of the exposure variable, *x*_1_) is equal to the estimated coefficient for *x*_1_ in the composite UR model.
- **Property (iii):** The estimated coefficient for each *x_i_* in its individual standard regression model (i.e. for designated exposure *x_i_*) is equal to the estimated coefficient for the corresponding UR term $e_{\mathrm{xi}}$ in the composite UR model.

From a causal inference perspective, only Properties (ii) and (iii) are meaningful, since the focus is on individual coefficient estimates as opposed to predicted outcomes. Nevertheless, we evaluate all three properties in Sections 4 through 6, and leave discussion of interpretational issues until later in the paper (§8).

## 4 UR models: No confounders (Figure 1(a))

Before considering any additional confounding variables, we first consider the straightforward scenario depicted in Figure 1(a). We provide: definitions of the standard regression models, UR terms, and UR models (§4.1); an analysis of UR models within a causal framework (§4.2); and arguments for why Properties (i)–(iii) are upheld (§4.3).

### 4.1 Definitions

We define the ordinary least-squares (OLS) regression model y^S(i) for estimating the total causal effect of each measurement of the exposure variable *x_i_* (for 1≤i≤k) on *y* as:

(5) y^S(i)=α^0(i)+α^x1(i)x1+α^x2(i)x2+…+α^xi(i)xi

A visual depiction of equation (5) is given in Figure 1(b). Because the relationship between each *x_i_* and *y* is confounded by all previous measurements of *x* (i.e. $x_{1},\ldots ,x_{i-1}$), these covariates must be adjusted for. However, as discussed in Section 1, only the coefficient of the last/most recent measurement of *x* (i.e. α^xi(i)) may be interpreted as a total causal effect.

To create UR terms according to the process established by Keijzer-Veen et al.,^9^ each measurement of the exposure *x_i_* is regressed on all previous measurements of*x* (for 2≤i≤k):

(6) xi=γ^0(i)+γ^x1(i)x1+γ^x2(i)x2+…+γ^x(i-1)(i)xi-1+exi

The UR term $e_{\mathrm{xi}}$ thus represents the difference between the actual value of *x_i_* and the value of *x_i_* as predicted by all previous measurements of *x*.

Lastly, we define the UR model y^UR(i) (for 1≤i≤k), which represents the outcome *y* as function of the initial value of the exposure *x*_1_ and subsequent ‘unexplained’ increases $e_{x2},\ldots ,e_{\mathrm{xi}}:$

(7) y^UR(i)=λ^0(i)+λ^x1(i)x1+λ^ex2(i)ex2+…+λ^exi(i)exi

The composite UR model y^UR(k) thus represents the outcome *y* as function of the initial value of the exposure *x*_1_ and *all* subsequent ‘unexplained’ increases $e_{x2},\ldots ,e_{\mathrm{xk}}$. The UR modelling process is summarised in Figure 1(c), depicting k-1 regressions of *x_i_* on $x_{1},\ldots ,x_{i-1}$ (equation (6)) and one composite UR regression model (equation (7), with i=k).

### 4.2 A causal framework

Within the causal framework provided by Figure 1(a), the unique properties of UR models can be visualised. If we were naively to model $x_{1},x_{2},\ldots ,x_{k}$ simultaneously, only the coefficient of the final measurement *x_k_* could be interpreted as a total causal effect on *y*; the coefficients of $x_{1},\ldots ,x_{k-1}$ would represent only the direct effects of each measurement on *y*, because all future measurements would fully mediate the respective relationship and all backdoor paths^1^ would be blocked by preceding measurements. However, by modelling $x_{1},e_{x2},\ldots ,e_{\mathrm{xk}}$ (as in a UR model), we encounter no mediation problems due to the fact that, by construction, the UR terms remain wholly independent of the other terms in the model. In fact, by placing the UR model in a causal framework, we are able to see that the UR terms $e_{x2},\ldots ,e_{\mathrm{xk}}$ are essentially instrumental variables (IVs)^42^ for $x_{2},\ldots ,x_{k}$, respectively, which have been produced by the modelling process (Note: The process has similarities with the two-stage least squares regression method,^43^ a form of instrumental variable analysis commonly encountered in economics research).

All techniques based on linear regression, including UR models, assume that the causal relationships between variables are linear functions. If that is the case, we may parameterise a DAG (as in Figure 1(a)) by assigning a single coefficient to every arrow and assuming all variables to have a variance of one. The method of path coefficients^44^ then allows us to determine the ‘true’ total causal effects in the data generating process. Take *x*_2_ as an example, where k=3. The total effect of *x*_2_ on *y* encompasses the direct effect from $x_{2}\to y$ and all indirect effects (of which there is only one in this scenario): $x_{2}\to x_{3}\to y$. We introduce the notation $p_{\mathrm{ba}}$ to represent the coefficient of the arrow a→b. Table 1 gives the total effects of *x*_2_ on *y* and of $e_{x2}$ on *y*, with both total effects decomposed into their respective direct and indirect effects. From Table 1, we see that the total effect of *x*_2_ on *y* is equal to the total effect of $e_{x2}$ on *y*; this is because there are no direct paths between $e_{x2}$ and *y*, and all indirect paths pass through *x*_2_ (with $p_{x_{2}e_{x2}}$ being equal to one, as in Figure 1(c)).

**Table 1.** Total effect of *x*_2_ on *y* estimated by a standard regression model compared to total effect of $e_{x2}$ on *y* estimated by an equivalent UR model (Figure 1(a), with k=3).

Exposure	Path		Effect size	Total effect
*x*_2_				
	Direct:	$x_{2}\to y$	$p_{{\mathrm{yx}}_{2}}$	$p_{{\mathrm{yx}}_{2}}+p_{x_{3}x_{2}}\cdot p_{{\mathrm{yx}}_{3}}$
	Indirect:	$x_{2}\to x_{3}\to y$	$p_{x_{3}x_{2}}\cdot p_{{\mathrm{yx}}_{3}}$	
$e_{x2}$				
	Direct:	n/a		$p_{{\mathrm{yx}}_{2}}+p_{x_{3}x_{2}}\cdot p_{{\mathrm{yx}}_{3}}$
	Indirect:	$e_{x2}\to x_{2}\to y$	$p_{x_{2}e_{x2}}\cdot p_{{\mathrm{yx}}_{2}}$	
		$e_{x2}\to x_{2}\to x_{3}\to y$	$p_{x_{2}e_{x2}}\cdot p_{x_{3}x_{2}}\cdot p_{{\mathrm{yx}}_{3}}$	

### 4.3 Covariate orthogonality and Properties (i)–(iii)

In addition to the graph-based approach in the preceding section, we are able to prove mathematically that Properties (i)–(iii) are upheld for the scenario given in Figure 1(a). In summary, these properties are:

- **Property (i):** y^S(k)=y^UR(k)
- **Property (ii):** α^x1(1)=λ^x1(k)
- **Property (iii):** α^xi(i)=λ^exi(k)

Equations (5) to (7) are summarised in Table 2; the standard regression models y^s(i) (for 1≤i≤k) and composite UR model y^UR(k) (in which the UR terms have been produced via the regression of each measurement of *x* on all previous measurements, as in equation (5)) contained therein are guaranteed to satisfy Properties (i)–(iii). These properties of UR models rely crucially on all UR terms $e_{x2},\ldots ,e_{\mathrm{xk}}$ being orthogonal to all other covariates in the composite UR model y^UR(k).

**Table 2.** For the scenario depicted in Figure 1(a), the standard regression model y^S(i) necessary for estimating the total causal effect of each exposure *x_i_* on *y*, and the corresponding UR model y^UR(i), for 1≤i≤k.

	Standard regression model y^S(i)	UR model y^UR(i)
i=1:	α^0(1)+α^x1(1)x1	λ^0(1)+λ^x1(1)x1
i=2:	α^0(2)+α^x1(2)x1+α^x2(2)x2	λ^0(2)+λ^x1(2)x1+λ^ex2(2)ex2
⋮	⋮	⋮
i=k:	α^0(k)+α^x1(k)x1+α^x2(k)x2+…+α^xk(k)xk	λ^0(k)+λ^x1(k)x1+λ^ex2(k)ex2+…+λ^exk(k)exk

We illustrate this property, and explain how it is exploited to ensure Properties (i)–(iii) are upheld. Formal proofs are provided in online supplementary Appendix 1.

In Table 2, note that each regression model (for both the standard and UR methods) contains one more covariate than the model preceding it. In the column of standard regression models, each row contains an additional *x_i_* term; in the column of UR models, each row contains an additional $e_{\mathrm{xi}}$ term.

Typically, the inclusion of an additional covariate in a regression model changes the coefficient(s) estimated for other covariates because their covariance would be nonzero. For example, the addition of *x*_2_ in y^s(2) will undoubtedly change the estimated coefficient for *x*_1_ in y^S(2) compared to y^S(1), because *x*_1_ and *x*_2_ are two measurements of the same variable and thus will have a nonzero covariance (i.e. correlation ≠ 0). This nonzero covariance is what is exploited by adjustment for confounders – if two covariates did not covary, then adjustment would not be necessary in the first place.

However, a UR model upholds Properties (ii) and (iii) specifically because its covariates do not covary. The addition of $e_{x2}$ in y^UR(2) does not change the estimated coefficient for *x*_1_ in y^UR(2) compared to y^r(1) because *x*_1_ and $e_{x2}$ are orthogonal (i.e. correlation = 0). This orthogonality is ensured as an artefact of OLS regression; because $e_{x2}$ represents the residual term from the regression of *x*_2_ on *x*_1_ by definition (equation (6)), it is guaranteed to be orthogonal to *x*_1_.

In fact, it can easily be shown that all UR terms $e_{x2},\ldots ,e_{\mathrm{xk}}$ are orthogonal to one another by construction. For any UR term $e_{\mathrm{xi}}$, it holds that $e_{\mathrm{xi}}$ is orthogonal to $x_{1},\ldots ,x_{i-1}$. Because preceding UR terms $e_{x2},\ldots ,e_{x(i-1)}$ are themselves linear combinations of $x_{1},\ldots ,x_{i-1}$ (equation (6)), it follows that $e_{\mathrm{xi}}$ is orthogonal to $e_{x2},\ldots ,e_{x(i-1)}$, for 2≤i≤k. Using this information, we can easily conclude that the addition of subsequent UR terms in the set of UR models in Table 2 will leave the coefficients of all other covariates unchanged. Thus, it only remains to be shown that the estimated coefficients for *x*_1_ and the UR terms $e_{x2},\ldots ,e_{\mathrm{xk}}$ are themselves equivalent to the coefficients for $x_{1},x_{2},\ldots ,x_{k}$ as estimated in their individual standard regression models, respectively.

#### Property (i):

First, it must be noted that each UR model is nothing more than a reparameterisation of the corresponding standard regression model (i.e. y^S(i)=y^UR(i) for each row in Table 2). Each standard regression model y^S(i) represents *y* as a function of $x_{1},\ldots ,x_{i}$. In contrast, each UR model y^UR(i) represents *y* as a function of $x_{1},e_{x2},\ldots ,e_{\mathrm{xi}}$. However, $e_{\mathrm{xi}}$ is itself a function of $x_{1},\ldots ,x_{i}$ (equation (5)), and thus it follows that the UR model y^UR(i) itself is also a function of $x_{1},\ldots ,x_{i}$. Because y^S(i) and y^UR(i) are both functions of the same covariates, it follows that y^S(k)=y^UR(k), thereby satisfying Property (i).

#### Property (ii):

It is trivially true that the coefficients estimated for *x*_1_ in the first standard regression model y^S(1) and corresponding UR model y^UR(1) will be equal (i.e. α^x1(1)=λ^x1(1)) because the models are themselves equivalent. All subsequent UR terms $e_{x2},\ldots ,e_{\mathrm{xk}}$ are orthogonal to *x*_1_ and to one another; therefore, it follows that the estimated coefficient of *x*_1_ will be equivalent for all UR models in Table 1 (i.e. λ^x1(1)=λ^x1(2)=…=λ^x1(k)). This ensures that the coefficient of *x*_1_ in y^S(1) (which represents the total effect of *x*_1_ on *y*) will be unchanged in the composite UR model y^UR(k) (i.e. α^x1(1)=λ^x1(k)).

#### Property (iii):

Lastly, we can show that the coefficient for $e_{\mathrm{xi}}$ (i.e. λ^exi(i)) in a UR model is equal to the estimated total effect of *x_i_* (i.e. α^xi(i)) in the corresponding standard regression model. To this end, we consider the following standard regression and corresponding UR models, respectively:

y^S(i)=α^0(i)+α^x1(i)x1+α^x2(i)x2+…+α^xi(i)xiy^UR(i)=λ^0(i)+λ^x1(i)x1+λ^ex2(i)ex2+…+λ^exi(i)exi

We may set these two equations equal to one another (due to Property (i)), substitute the expansions for $e_{x2},\ldots ,e_{\mathrm{xi}}$ (equation (5)) into the UR model and rearrange, thereby producing:

(8) α^ 0(i)+α^x1(i)x1+α^x2(i)x2+…+α^xi(i)xi=λ^0(i)+λ^x1(i)x1+λ^ex2(i)ex2+…+λ^exi(i)exi=λ^0(i)+λ^x1(i)x1+λ^ex2(i)[-γ^0(2)-γ^x1(2)x1+x2]+…+λ^exi(i)[-γ^0(i)-γ^x1(i)x1-γ^x2(i)x2-…-γ^x(i-1)(i)xi-1+xi]=[λ^0(i)-λ^ex2(i)γ0(2)-…-λ^exi(i)γ0(i)]+[λ^x1(i)-λ^ex2(i)γx1(2)-…-λ^exi(i)γx1(i)]x1+[λ^ex2(i)-λ^ex3(i)γx2(3)-…-λ^exi(i)γx2(i)]x2+…+[λ^exi(i)]xi

From equation (8) above, it becomes clear that the coefficients for *x_i_* in y^S(i) and $e_{\mathrm{xi}}$ in y^UR(i) are equal (i.e. α^xi(i)=λ^exi(i)). Again, we invoke the property of orthogonality to conclude that the estimated coefficient for $e_{\mathrm{xi}}$ will be equivalent for all UR models in Table 2 (i.e. λ^exi(1)=λ^exi(2)=…=λ^exi(k)). This ensures that the coefficient of $e_{\mathrm{xi}}$ in y^S(i) (which represents the total effect of *x_i_* on *y*) will be unchanged in the composite UR model y^UR(k) (i.e. α^xi(i)=λ^exi(k)).

## 5 UR models: Time-invariant confounder (Figure 2(a))

We next consider the scenario in Figure 2(a), in which a time-invariant covariate *m* confounds the relationship between $x_{1},x_{2},\ldots ,x_{k}$ and *y*. This section is structured similarly to the preceding one. We provide: definitions of the standard regression models, UR terms, and UR models, all adjusted for the confounder *m* based upon the DAG in Figure 2(a) (§5.1); an analysis of UR models within a causal framework (§5.2); arguments for why Properties (i)–(iii) are upheld when the defined adjustments for *m* have been made (§5.3); and a discussion regarding the implications of insufficient adjustment for *m* (§5.4).

### 5.1 Definitions (with correct adjustment for m)

Using the DAG in Figure 2(a) as guidance, we extend the original definitions of the standard regression models, UR terms, and UR models (equations (5) to (7), respectively) to properly account for the confounding effect of *m*, a time-invariant covariate.

We define the OLS regression model y^S(i) for estimating the total causal effect of each measurement of the exposure variable *x_i_* (for 1≤i≤k) on *y* as:

(9) y^S(i)=α^0(i)+α^m(i)m+α^x1(i)x1+α^x2(i)x2+…+α^xi(i)xi

Because the relationship between each *x_i_* and *y* is confounded by all previous measurements of *x* (i.e. $x_{1},\ldots ,x_{i-1}$) and *m*, these covariates must be adjusted for to obtain an inferentially unbiased estimate of the total causal effect of each measurement of the exposure. As previously, only the coefficient of the last/most recent measurement of *x* (i.e. α^xi(i)) may be interpreted as a total causal effect.

We further extend the process of Keijzer-Veen et al.^9^ to create UR terms for this scenario. As is evident, the relationship between each measurement of the exposure variable *x_i_* and all previous measurements $x_{1},\ldots ,x_{i-1}$ is confounded by *m* (for 2≤i≤k); thus, adjustment for *m* is necessary:

(10) xi=γ^0(i)+γ^m(i)m+γ^x1(i)x1+γ^x2(i)x2+…+γ^x(i-1)(i)xi-1+exi

Therefore, the UR term $e_{\mathrm{xi}}$ represents the difference between the actual value of *x_i_* and the value of *x_i_* as predicted by all previous measurements $x_{1},\ldots ,x_{i-1}$, *adjusted for the confounding effect of* *m*.

Finally, we define the UR model y^UR(i) (for 1≤i≤k); this model must be also be adjusted for *m*, since *m* confounds the relationship between *x*_1_ and y:

(11) y^UR(i)=λ^0(i)+λ^m(i)m+λ^x1(i)x1+λ^ex2(i)ex2+…+λ^exi(i)exi

The composite UR model y^UR(k) thus represents the outcome *y* as function of the initial value of the exposure *x*_1_, all subsequent ‘unexplained’ increases $e_{x2},\ldots ,e_{\mathrm{xk}}$, and the time-invariant confounder *m*.

As in the preceding section, visual depictions of the previous equations are provided, with Figure 2(b) corresponding to equation (8) and Figure 2(c) corresponding to equation (8) and equation (9) (with i=k).

### 5.2 A causal framework

We may easily extend the reasoning from the previous scenario (§4.2) to explain why the UR model (equation (11)) satisfies Properties (i)–(iii) before resorting to mathematics, by considering the diagram in Figure 2(a) as a path diagram. A regression model containing all of $m,x_{1},x_{2},\ldots ,x_{k}$ (as in equation (9)) would only allow for the interpretation of the coefficient of *x_k_* as a total causal effect on *y*; the coefficients of $x_{1},\ldots ,x_{k-1}$ would represent only the direct effects of each measurement on *y*, because all future measurements would mediate the respective relationship and all backdoor paths would be blocked by preceding measurements (including *m*). Within the UR model, the independence of all UR terms $e_{x2},\ldots ,e_{\mathrm{xk}}$ ensures no mediating paths are blocked, and the only backdoor path between *x*_1_ and *y* is blocked by *m*.

### 5.3 Covariate orthogonality and Properties (i)–(iii)

In addition to the graph-based approach in the preceding section (§5.2), we are able to illustrate mathematically that adjustment for *m* both when generating each UR term $e_{\mathrm{xi}}$ (equation (10)) and in the composite UR model (Eq.11) will result in Properties (i)–(iii) being satisfied. Note that the scenario depicted in Figure 2(a) is nearly indistinguishable, both visually and mathematically, from the scenario in Figure 1(a). The confounder *m* (which affects *y* and all measurements of *x*) could be reimagined as variable *x*_0_; viewed in this way, the need for its adjustment becomes clear and the proofs from the previous section apply with only minor notational adjustments. Even though a distinction must be drawn between exposure variables and confounding variables within a causal framework, OLS regression treats both equivalently (i.e. as ‘independent variables’). Therefore, we give a brief outline only of how the adjustments deemed necessary by the causal diagram in Figure 2(a) will result in Properties (i)–(iii) being upheld and attach the formal mathematical proofs in online supplementary Appendix 2.

Equations (9) to (11), which are summarised in Table 3, are guaranteed satisfy Properties (i)–(iii). As in the previous scenario (§4.3), each regression model (for both the standard and UR methods) in Table 3 contains one more covariate than the model preceding it – an additional *x_i_* term in the column of standard regression models, and an additional $e_{\mathrm{xi}}$ term in the column of UR models. Proofs for the previous scenario relied on the property of each UR term being orthogonal to all preceding terms in the model. Adjustment for *m* when generating each UR term $e_{\mathrm{xi}}$ (equation (10)) guarantees that this property will be upheld, because it ensures that $e_{\mathrm{xi}}$ is orthogonal to *m* in addition to $e_{x1},\ldots ,e_{x(i-1)}$; this cannot be guaranteed without explicit adjustment for *m*. Furthermore, adjustment for *m* in each UR model in Table 3 ensures that y^S(i)=y^UR(i) for each row in Table 3.

**Table 3.** For the scenario depicted in Figure 2(a), the standard regression model y^S(i) necessary for estimating the total causal effect of each exposure *x_i_* on *y*, and the corresponding UR model y^UR(i), for 1≤i≤k.

	Standard regression model y^S(i)	UR model y^UR(i)
i=1:	α^0(1)+α^m(1)m+α^x1(1)x1	λ^0(1)+λ^m(1)m+λ^x1(1)x1
i=2:	α^0(2)+α^m(2)m+α^x1(2)x1+α^x2(2)x2	λ^0(2)+λ^m(2)m+λ^x1(2)x1+λ^ex2(2)ex2
⋮	⋮	⋮
i=k:	α^0(k)+α^m(k)m+α^x1(k)x1+α^x2(k)x2+…+α^xk(k)xk	λ^0(k)+λ^m(k)m+λ^x1(k)x1+λ^ex2(k)ex2+…+λ^exk(k)exk

### 5.4 Incorrect adjustment for m

We have used the causal diagram in Figure 2(a) to argue for the necessity of adjusting for a time-invariant confounder *m* during both stages of the UR modelling process, and have demonstrated how such adjustments will produce a composite UR model that satisfies Properties (i)–(iii), as Keijzer-Veen et al. intended. We now consider the implications of insufficient adjustment.

Without adjustment for *m* when generating each UR term $e_{\mathrm{xi}}$, the coefficients of $x_{1},\ldots ,x_{i-1}$ (i.e. γ^xi(j), for 1≤i≤k-1 and 1≤j≤k) and the UR term will absorb the effect of the omitted variable *m* on *x_i_*, thereby biasing the total effect of $e_{\mathrm{xi}}$ estimated within the UR model (so-called ‘omitted variable bias’). Further, it is evident that *m* confounds the relationship between *x*_1_ and *y*, so that failure to adjust for *m* in the composite UR model will produce different predicted outcome values and bias the estimated coefficient of *x*_1_.

## 6 UR models: Time-varying confounder (Figure 3(a))

Finally, we consider the scenario in Figure 3(a), in which a time-varying covariate $m_{1},m_{2},\ldots ,m_{k}$ confounds the relationship between $x_{1},x_{2},\ldots ,x_{k}$ and *y*.

In this section, we again provide: definitions of the standard regression models, UR terms, and UR models, all adjusted for the confounder $m_{1},m_{2},\ldots ,m_{k}$ based upon the DAG in Figure 3(a) (§6.1); an analysis of UR models within a causal framework (§6.2); arguments for why Properties (i)–(iii) are upheld when the defined adjustments for $m_{1},m_{2},\ldots ,m_{k}$ have been made (§6.3); and a discussion regarding the implications of insufficient adjustment for $m_{1},m_{2},\ldots ,m_{k}$ (§6.4).

### 6.1 Definitions (with correct adjustment for $m_{1},m_{2},\ldots ,m_{k}$)

Using the DAG in Figure 3(a), we extend the original definitions of the standard regression models, UR terms, and UR models (equations (5) to (7), respectively) to properly account for the confounding effect of $m_{1},m_{2},\ldots ,m_{k}$, a time-varying covariate.

We define the OLS regression model y^S(i) for estimating the total causal effect of each measurement of the exposure variable *x_i_* (for 1≤i≤k) on *y* as:

(12) y^S(i)=α^0(i)+α^m1(i)m1+α^x1(i)x1+…+α^mi(i)mi+α^xi(i)xi

The relationship between each *x_i_* and *y* is not only confounded by all previous values of the exposure $x_{1},\ldots ,x_{i-1}$ but also by the current measurement and all previous measurements of the confounder $m_{1},\ldots ,m_{i}$. Therefore, adjustment for $m_{1},\ldots ,m_{i},x_{1},\ldots ,x_{i-1}$ is necessary to obtain an inferentially unbiased estimate of the total causal effect of each measurement of the exposure. We reiterate that only the coefficient of the last/most recent measurement of *x* (i.e. α^xi(i)) may be interpreted as a total causal effect.

Extending the process of Keijzer-Veen et al.^9^ to create UR terms for each measurement of the exposure *x_i_* in this scenario necessitates adjustment for the current measurement and all previous measurements of the confounder $m_{1},m_{2},\ldots ,m_{i}$ (for 2≤i≤k), since these variables confound the relationship between each measurement of the exposure variable *x_i_* and all previous measurements $x_{1},\ldots ,x_{i-1}$, i.e.:

(13) xi=γ^0(i)+γ^m1(i)m1+γ^x1(i)x1+…+γ^m(i-1)(i)mi-1+γ^x(i-1)(i)xi-1+γ^mi(i)mi+exi

In this way, $e_{\mathrm{xi}}$ represents the difference between the observed value of *x_i_* and the value of *x_i_* as predicted by all previous measurements $x_{1},\ldots ,x_{i-1}$, *adjusted for the confounding effects of* $m_{1},m_{2},\ldots ,m_{i}$.

As we have demonstrated previously (§4.3, §5.3), UR models rely upon the orthogonality of terms in the composite UR model. This necessitates the creation of UR terms $e_{\mathrm{mi}}$ for each measurement of the time-varying confounding variable *m_i_* (for 2≤i≤k) in a similar manner to that of the UR terms $e_{\mathrm{xi}}$ (equation (13)). Each $e_{\mathrm{mi}}$ is derived from the OLS regression of *m_i_* on all previous values of the confounder $m_{1},\ldots ,m_{i-1}$, as well as all previous values of the exposure $x_{1},x_{2},\ldots ,x_{i-1}$ which confound this relationship:

(14) mi=η^0(i)+η^m1(i)m1+η^x1(i)x1+…+η^m(i-1)(i)mi-1+η^x(i-1)(i)xi-1+emi

Thus, $e_{\mathrm{mi}}$ has a similar interpretation to the original UR term $e_{\mathrm{xi}}$, in that it represents the part of *m_i_* unexplained by all previous values $m_{1},\ldots ,m_{i-1}$, *adjusted for the confounding effects of* $x_{1},\ldots ,x_{i-1}$.

Lastly, we define the UR model y^UR(i) (for 1≤i≤k) as a function of the initial value of the confounder *m*_1_ and its subsequent ‘unexplained’ increases $e_{m2},\ldots ,e_{\mathrm{mi}}$, and the initial value of the exposure *x*_1_ and its subsequent ‘unexplained’ increases $e_{x2},\ldots ,e_{\mathrm{xi}}:$

(15) y^UR(i)=λ^0(i)+λ^m1(i)m1+λ^x1(i)x1+λ^em2(i)em2+λ^ex2(i)ex2+…+λ^emi(i)emi+λ^exi(i)exi

As previously, visual depictions of these equations are provided. Figure 3(b) corresponds to the standard regression models given by equation (12); Figure 3(c) corresponds to the k-1 regressions of *x_i_* on all preceding measurements of *x* and *m* (equation (13)), the k-1 regressions of *m_i_* on all preceding measurements of *x* and *m* (equation (14)), and one composite UR regression model (equation (15), with i=k).

### 6.2 A causal framework

The similarities amongst the three causal scenarios depicted in Figures 1(a), 2(a), and 3(a) are evident, and shed light on how the reasoning from the previous scenarios (§4.2 and §5.2) can be extended to demonstrate why the UR model in equation (15) satisfies Properties (i)–(iii). In a regression model containing all of $m_{1},\ldots ,m_{k},x_{1},\ldots ,x_{k}$ (as in equation (12), with i=k), only the coefficient of *x_k_* could be interpreted as a total causal effect on *y*; the coefficients of $x_{1},\ldots ,x_{k-1}$ may only be interpreted as the direct effects of each measurement of the exposure on *y*, because all future measurements of both *x* and *m* would fully mediate the respective relationship and all preceding measurements of *x* and *m* would block all backdoor paths. Within the UR model, however, the independence of all UR terms for both the exposure (i.e. $e_{x2},\ldots ,e_{\mathrm{xk}}$) and confounder (i.e. $e_{m2},\ldots ,e_{\mathrm{mk}}$) ensures no mediating paths are blocked, and the only backdoor path between *x*_1_ and *y* is blocked by *m*_1_.

### 6.3 Covariate orthogonality and Properties (i)–(iii)

In addition to the graph-based approach in the preceding section (§6.2), we can illustrate mathematically that the standard regression models y^S(i) (equation (12)), UR terms for measurements of the exposure (equation (13)) and confounder (equation (14)), and composite UR model y^UR(k) (equation (15), with i=k) satisfy Properties (i)–(iii). Although seemingly more complex, the scenario depicted in Figure 3(a) also has very little to distinguish it from the scenarios in Figures 1(a) and 2(a). The confounder *m*_1_, being the only exogenous node on the graph, could be imagined as variable *x*_0_, with all nodes subsequent to *x*_1_ having an associated UR term. Viewed as such, the necessity of adjusting for *m*_1_ and creating UR terms for both the exposure and the time-varying confounder becomes apparent, as the causal diagram in Figure 3(a) is equivalent to that of Figure 2(a) with minor notational adjustments. Therefore, we provide only a brief outline of how the adjustments deemed necessary by the causal diagrams in Figure 3(a) will result in Properties (i)–(iii) being upheld; formal mathematical proofs are provided in online supplementary Appendix 3.

Equations (12) to (15) are summarised in Table 4 and are guaranteed to satisfy Properties (i)–(iii). In contrast to previous scenarios (§4.3 and §5.3), each regression model (for both the standard and UR models) contains *two* more covariates than the model preceding it. In the column of standard regression models, each row contains an additional *x_i_* and *m_i_* term; in the column of UR models, each row contains an additional $e_{\mathrm{xi}}$ and $e_{\mathrm{mi}}$ term. Thus, for Properties (i)–(iii) to be upheld in in each UR model y^UR(i), these two additional terms must be orthogonal to one another and to all preceding terms.

**Table 4.** For the scenario depicted in Figure 3(a), the standard regression model y^S(i) necessary for estimating the total causal effect of each exposure *x_i_* on *y*, and the corresponding UR model y^UR(i), for 1≤i≤k.

	Standard regression model y^S(i)	UR model y^UR(i)
i=1:	α^0(1)+α^m1(1)m1+α^x1(1)x1	λ^0(1)+λ^m1(1)m1+λ^x1(1)x1
i=2:	α^0(2)+α^m1(2)m1+α^x1(2)x1+γ^m2(2)m2+α^x2(2)x2	λ^0(2)+λ^m1(2)m1+λ^x1(2)x1+λ^em2(2)em2+λ^ex2(2)ex2
⋮	⋮	⋮
i=k:	α^0(k)+α^m1(k)m1+α^x1(k)x1+α^m2(k)m2+α^x2(k)x2+…+α^mk(k)mk+α^xk(k)xk	λ^0(k)+λ^m1(k)m1+λ^x1(k)x1+λ^em2(k)em2+λ^ex2(k)ex2+…+λ^emk(k)emk+λ^exk(k)exk

Proving this is relatively straightforward. For any UR term $e_{\mathrm{mi}}$ for the confounder, it holds that $e_{\mathrm{mi}}$ is orthogonal to $m_{1},\ldots ,m_{i-1},x_{1},\ldots ,x_{i-1}$ by construction (equation (14)). Because preceding UR terms $e_{x2},\ldots ,e_{x(i-1)}$ (equation (13)) and $e_{m2},\ldots ,e_{m(i-1)}$ (equation (14)) may be expressed as linear combinations of $m_{1},\ldots ,m_{i},x_{1},\ldots ,x_{i-1}$, it follows that $e_{\mathrm{mi}}$ is orthogonal to $e_{m2},\ldots ,e_{m(i-1)},e_{x2},\ldots ,e_{x(i-1)}$. Furthermore, for any UR term $e_{\mathrm{xi}}$ for the exposure, it holds that $e_{\mathrm{xi}}$ is orthogonal to $m_{1},\ldots ,m_{i},x_{1},\ldots ,x_{i-1}$ by construction (equation (13)). Because preceding UR terms $e_{x2},\ldots ,e_{x(i-1)}$ (equation (13)) and $e_{m2},\ldots ,e_{\mathrm{mi}}$ (equation (14)) may be expressed as linear combinations of $m_{1},\ldots ,m_{i},x_{1},\ldots ,x_{i-1}$, it follows that $e_{\mathrm{xi}}$ is orthogonal to $e_{m2},\ldots ,e_{\mathrm{mi}},e_{x2},\ldots ,e_{x(i-1)}$. Thus, we are able to conclude that $e_{\mathrm{mi}}$ and $e_{\mathrm{xi}}$ are orthogonal to one another and to all preceding terms in for any UR model y^UR(i); adjustment for all causally preceding measurements of both *m* and *x* when generating UR terms for both the confounder and the exposure ensures this orthogonality.

### 6.4 Incorrect adjustment for $m_{1},m_{2},\ldots ,m_{k}$

The DAG in Figure 3(a) demonstrates the necessity of adjusting for a time-varying confounder $m_{1},m_{2},\ldots ,m_{k}$ in the manner described in Section 6.1, and we have demonstrated how such adjustments will produce a composite UR model that satisfies Properties (i)–(iii). The implications of incorrect adjustment for a time-varying confounder $m_{1},m_{2},\ldots ,m_{k}$ in a UR model are similar to those of incorrect adjustment for a time-invariant confounder *m*, which were previously outlined in Section 5.4. Without adjustment for any of $m_{1},\ldots ,m_{i}$ when constructing each UR term for the exposure $e_{\mathrm{xi}}$, the coefficients of $x_{1},\ldots ,x_{i-1}$ (i.e. γ^xi(j), for 1≤i≤(k-1) and 1≤j≤k) and the UR term will absorb the effect of each omitted variable on *x_i_*; this will result in the coefficient estimated for each $e_{\mathrm{xi}}$ in the composite UR model being unequal to the total effect of *x_i_* in its corresponding standard regression model.

The requirement of orthogonal covariates within the composite UR model also sheds light on the necessity for generating UR terms $e_{m2},e_{m3},\ldots ,e_{\mathrm{mk}}$ for measurements of a time-varying confounder, if present. We might easily imagine a scenario in which we considered only the original covariates $m_{1},m_{2},\ldots ,m_{k}$ in the UR model. In such a scenario, the terms would remain correlated with each other and with *x*_1_; therefore, the inclusion of subsequent *m* terms in the UR model would necessarily change the coefficient estimates for *x*_1_ and all other covariates.

## 7 UR model interpretation

Having demonstrated that confounder adjustment within UR models is possible, we consider the claim^9^ that UR models offer additional insight via the coefficients for each UR term $e_{\mathrm{xi}}$ (e.g. λ^exi(k) in equation (7), for 2≤i≤k) into the effect of *x_i_* increasing *more than expected* upon *y*.

Consider again the simple example with two longitudinal measurements of a continuous exposure *x* (i.e. *x*_1_ and *x*_2_), outcome *y*, and no additional confounders (i.e. Figure 1(a), with k=2); the standard regression model (with *x*_2_ as the specified exposure variable) and ‘equivalent’ UR model are given below, respectively:

y^S(2)=α^0(2)+α^x1(2)x1+α^x2(2)x2y^UR(2)=λ^0(2)+λ^x1(2)x1+λ^ex2(2)ex2

It has been shown (§4.3) that α^x2(2) and λ^ex2(2) are equal, yet α^x2(2) is interpreted as the total effect of a one-unit increase in *x*_2_ on *y*, whereas λ^ex2(2) is (supposedly) interpreted as the total effect of a one-unit *higher than expected* increase in *x*_2_ on *y*. If these two variables truly are distinct, their regression coefficients should likewise be distinct. This issue has also been addressed by Tu and Gilthorpe,^11^ who have argued that the two coefficients are equivalent because adjustment for *x*_1_ in y^S(2) amounts to testing the relation between *y* and the part of *x*_2_ *unexplained by* *x*_1_ (i.e. the unexplained residual). In fact, the two coefficients are equal simply because they mean the same thing. The UR model does not, therefore, offer any additional insight into the effect of higher than expected change in *x* on the outcome.^15^

We also raise a more philosophical point, which speaks to the need for any model to reflect accurately the underlying data-generation process of a given scenario. As an artefact of OLS regression, the UR terms will always be mathematically independent of the value of the initial measurement of the exposure and all subsequent measurements. This is unlikely to be an accurate representation of real-world exposure variables. Many of these, such as body size, exhibit a consistent, cumulative presence that is only manifest at the discrete time points at which it is measured; these measurements are thus distinct only as a result of the discretisation of time within the measurement processes adopted. Moreover, in auxological studies, the phenomenon of so-called compensatory (or ‘catch up’) growth has been well documented, with accelerated growth being observed in individuals who begin with a low value of some measure, e.g. birthweight.^45,46^ Therefore, however convenient and mathematically sound it may be to model data in a way that implies complete statistical independence amongst an exposure variable’s initial value and its subsequent measurements, this assumption is likely to be implausible and unrealistic for most biological and social variables of interest to epidemiologists. This is a weakness shared by all conditional approaches (of which UR models are one), which has led several authors^47^ to recommend that the results be considered alongside those produced by other methods, rather than in isolation.

## 8 Standard error reduction

Finally, we address an important consequence of the use of UR models; namely, that they underestimate the standard errors (SEs) of estimated coefficients, thereby resulting in artificial precision of estimated effect sizes. Although focus on *statistical* significance by way of *p*-values and confidence intervals is not in and of itself justifiable within a causal framework (as focus is effect size and likely *functional* significance, e.g. the absolute risk posed or the potential for substantive intervention), we consider it an important issue to address as a matter of clarity for researchers seeking to use UR models.

To demonstrate, we have simulated 1000 non-overlapping random samples of 1000 observations from a multivariate normal distribution based upon the DAG in Figure 1(a) with k=2, using the ‘dagitty’ package (v. 0.2–2)^4,48^ in R (v. 3.3.2).^49^ Each sample was used to create: (1) the two standard regression models necessary for estimating the total causal effect of each of $x_{1},x_{2}$ on *y* (equation (5)); (2) the UR term $e_{x2}$, derived by regressing *x*_2_ on *x*_1_ (equation (6)); and (3) the composite UR model in which *y* is regressed on *x*_1_ and $e_{x2}$ (equation (7)). For each standard regression model y^S(i) (for i=1,2), the reported SE of the regression coefficient for exposure *x_i_* is stored. For each composite UR model y^UR(2), the SE of the regression coefficient for each of $x_{1},e_{x2}$ is stored in two forms: (1) as reported in the UR model summary output; and (2) as estimated by bootstrapping 1000 samples and calculating the standard deviation of the distribution of estimated coefficients. Additional details relating to this simulation – including parameters and code – are located in online supplementary Appendix 4. (Note: The specific correlation structure and parameter values used to simulate the data are unimportant for the purposes of this demonstration).

By definition, the SE of an estimated regression coefficient is a point estimate of the standard deviation of an (infinitely) large sampling distribution of estimated regression coefficients. We have shown that standard regression and UR models elicit identical point estimates of the total causal effects of each measure of the longitudinal exposure (§4); from this, it follows that the associated SEs should themselves be equal.

**Figure 4.**
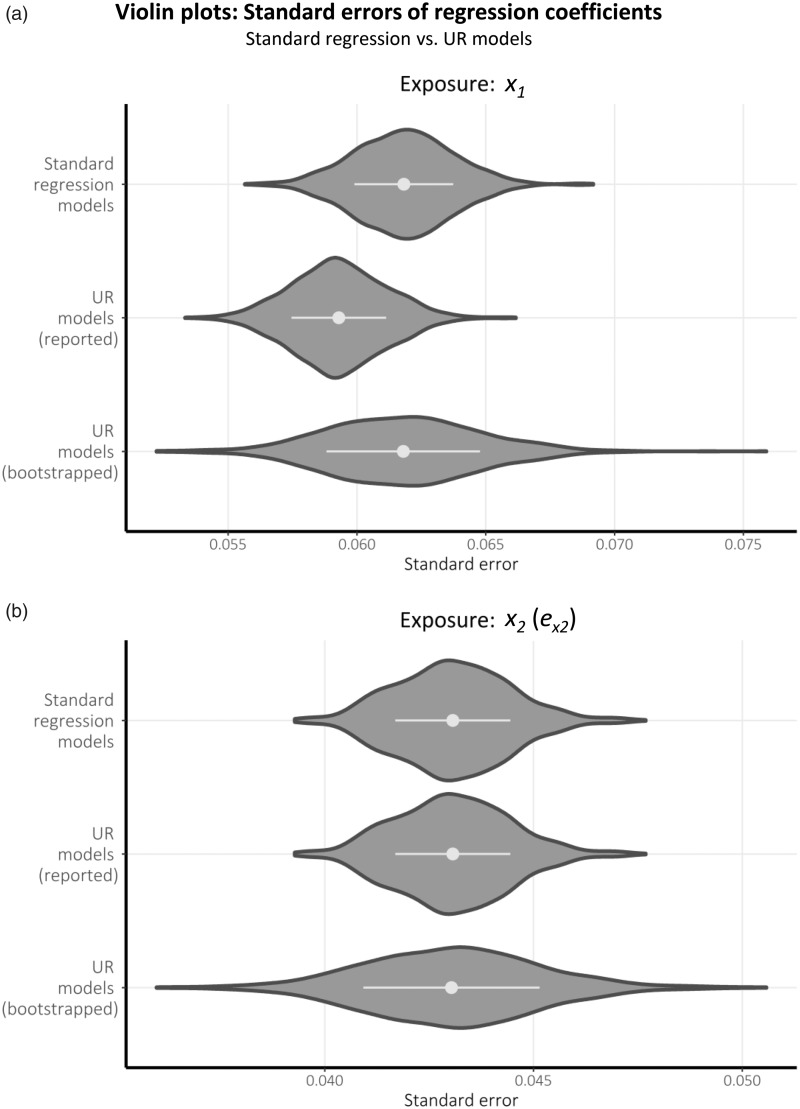
Violin plots comparing the standard errors associated with equivalent coefficients estimated in standard regression vs. UR models, for data simulated based upon the scenario depicted in Figure 1(a) (with *k*  = 2). Horizontal bars within each distribution represent the mean ± 1 standard deviation.

Violin plots of the SEs estimated for each coefficient representing a total causal effect across the 1000 simulations are displayed in Figure 4 for each method considered. As is evident, the *reported* SEs within the UR models are reduced in comparison to those within the first standard regression models (for designated exposure *x*_1_) and equal to those within the final standard regression models (for designated exposure *x*_2_). This demonstrates an apparent paradox: the coefficient values are equivalent, yet the associated SEs are unequal.

We argue that the apparent reduction in SEs achieved by using UR models is purely artefactual and arises from the explicit conditioning on future measurements of *x* within a UR model. In the standard regression analysis, the only information within the data that is used to inform SE estimation lies in the past (i.e. past measures of the exposure plus any confounders). In contrast, the UR modelling process generates (orthogonal) residuals for the entire exposure period and combines these into a single model, thereby using information within the data that is from both the past *and the future*. If we possessed data pertaining to any true independent causes of future measurements of the exposure, such a method would indeed be valid; however, the UR terms are simply estimated using prior measurements of the exposure. Moreover, due to the fact that they are estimates, the UR terms themselves contain additional variation that is not accommodated by traditional regression methods which assume covariates are measured without error. Consequently, the SEs of estimated causal effect derived from UR models are artefactually reduced and should not be inferred as robust. Indeed, when the SEs within the UR models are estimated via bootstrapping, they are similar to those within the standard regression models.

Comparing the two plots in Figure 4 offers clarity to this argument: (a) displays *differing* distributions of the reported SEs for the coefficient estimates of *x*_1_ (where conditioning on the future information given by *x*_2_ reduces the standard error in the UR model); whereas (b) displays the *same* distribution of the reported SEs for the coefficient estimates of *x*_2_ and $e_{x2}$ (where the standard regression model correctly exploits all prior information given by *x*_1_, as does the UR model). Although the magnitude of bias in estimated SEs is small in this simulated example, it will always be present due to the way in which UR models are constructed. Quantifying the magnitude of this bias is not trivial and is beyond the scope of the present study.

## 9 Conclusion

The mathematical appraisal of UR models that we have undertaken confirms that the method proposed by Keijzer-Veen et al.^9^ is capable of accommodating more than two longitudinal measurements of an exposure variable and demonstrates how adjustment for confounding variables should be made in this framework to uphold the property that the coefficients for the terms $x_{1},e_{x2},\ldots ,e_{\mathrm{xk}}$ estimated within a UR model are equal to the total effects for $x_{1},x_{2},\ldots ,x_{k}$ estimated by their respective standard regression models. This result will only be guaranteed to hold when adjustment for *all* confounding variables has been made at both stages in the UR modelling process (i.e. when generating UR terms for subsequent measurements of the exposure and in the composite UR model). From a statistical perspective, adjustment for all preceding variables (including confounders) ensures orthogonality amongst the covariates in a composite UR model. Therefore, when the potential confounder is time-varying, it is also necessary to generate UR terms for subsequent measurements of the confounder itself and include these in the final composite models used.

As our proofs only consider one confounding variable, the causal framework provided by DAGs should aid future researchers who wish to extend robustly UR models to situations involving multiple, possibly causally linked, time-invariant and time-varying confounders. Such a DAG will be useful in identifying confounders and establishing the temporal ordering of variables, thereby ensuring that all preceding variables are adjusted for when generating the necessary UR terms.

Although UR models can accommodate multiple measurements of an exposure variable in addition to confounding variables, we have concerns about their practical implementation. Although only one UR model need ultimately be presented, the necessity of generating orthogonal covariates for that UR model requires that many models be created; this has the potential to be quite substantial when multiple confounders are considered. For an exposure *x* measured at *k* points in time, the standard regression approach necessitates *k* separate models for estimating the total causal effect of each measurement on the outcome *regardless of the number of confounders*. In the case of one time-invariant confounder (§5), *k* models are also created (k-1 models to generate all UR terms and 1 composite UR model); for a time-varying confounder (§6), 2k-1 models are created (i.e. 2k-2 models to generate all UR terms and 1 composite UR model). The total number of models created by the UR process will always be either equal to or greater than the total number of models created by the standard regression process. If such a process offered real gains in insight into the scenario under consideration, it may indeed be worth it; however, UR models offer no additional insight compared to standard regression methods. Moreover, the inclusion of multiple covariates that are explicitly conditional on one another within the same model also results in artificially reduced standard error estimates, the extent of which has yet to be fully evaluated; the issue can be avoided by bootstrapping, but such a solution may be computationally intensive and require more programming skills than those necessary for implementing the built-in regression functionalities in statistical software packages. Previous research that has utilised UR models without undertaking sufficient adjustment for confounders and correcting SEs via bootstrapping should not be considered robust.

We therefore have strong reservations about the use and implementation of UR models within lifecourse epidemiology, and suggest that researchers considering using them should instead rely on standard regression methods, which produce the same results but are much less likely to be mis-specified and misleading. However, for researchers wishing to use these models, the hypothesised DAG or causal diagram should be presented so that any readers and/or reviewers can confirm that sufficient adjustment for confounders has been undertaken; moreover, SEs should be estimated via bootstrapping and not simply reported as in the model output, as these have the potential to be misleading. We support the recommendation of previous authors^47^ that additional analytical approaches should be considered alongside conditional approaches (e.g. UR models) in order to achieve robust causal conclusions. For example, multilevel, latent growth curve, and growth mixture models may be used to estimate the effects of growth across the lifecourse on a distal outcome, and are more flexible than standard regression methods.^5^ Moreover, the three G-methods^50,51^ are explicitly grounded in a causal framework and allow for the simultaneous consideration of multiple measurements of a longitudinally measured exposure, as well as time-varying confounding; these methods provide exciting avenues of research for lifecourse epidemiologists.

## Supplemental Material

Appendix -Supplemental material for Adjustment for time-invariant and time-varying confounders in ‘unexplained residuals’ models for longitudinal data within a causal framework and associated challenges Click here for additional data file. Supplemental material, Appendix for Adjustment for time-invariant and time-varying confounders in ‘unexplained residuals’ models for longitudinal data within a causal framework and associated challenges in Statistical Methods in Medical Research
