# Supplementary material for: Adjustment for time-invariant and time-varying confounders in ‘unexplained residuals’ models for longitudinal data within a causal framework and associated challenges
Source: Stat Methods Med Res. 2018 Feb 16;28(5):1347–64. doi: 10.1177/0962280218756158 (PMC6484949; doi:10.1177/0962280218756158)
Supplement: Appendix -Supplemental material for Adjustment for time-invariant and time-varying confounders in ‘unexplained residuals’ models for longitudinal data within a causal framework and associated challenges [file Appendix.pdf]

# 1. Appendix 1: UR models: No confounders

Proofs of Properties (i) – (iii) for the scenario depicted in Figure 1a (i.e. $k$ longitudinally measured exposure variables $x_1, x_2, \dots, x_k$ and one distal outcome $y$).

## 1.1. Definitions

### 1.1.1. Definition 1: Standard regression models

We define the ordinary least-squares (OLS) regression model $\hat{y}_S^{(i)}$ for each measurement of the exposure variable $x_i$, for $1 \le i \le k$. Because the relationship between $x_i$ and $y$ is confounded by all previous values of $x$ (i.e. $x_1, x_2, \dots, x_{i-1}$), we represent $y$ as a function of $1, x_1, x_2, \dots, x_i$:

$$\hat{y}_S^{(1)} = \hat{\alpha}_0^{(1)} + \hat{\alpha}_{x1}^{(1)} x_1$$

$$\hat{y}_S^{(2)} = \hat{\alpha}_0^{(2)} + \hat{\alpha}_{x1}^{(2)} x_1 + \hat{\alpha}_{x2}^{(2)} x_2$$

$$\vdots$$

$$\hat{y}_S^{(k)} = \hat{\alpha}_0^{(k)} + \hat{\alpha}_{x1}^{(k)} x_1 + \hat{\alpha}_{x2}^{(k)} x_2 + \dots + \hat{\alpha}_{xk}^{(k)} x_k \,. \qquad \text{(Eq.1)}$$

As discussed in Section 1, only the coefficient of the last/most recent measurement of $x$ (i.e. $\hat{\alpha}_{xi}^{(i)}$) may be interpreted as a total causal effect.

### 1.1.2. Definition 2: Unexplained residual (UR) terms

As established by Keijzer-Veen et al.[1], each UR term $e_{xi}$ is derived from the OLS regression of $x_i$ on all previous measurements of $x$ (i.e. $x_1, x_2, \dots, x_{i-1}$):

$$x_i = \hat{\gamma}_0^{(i)} + \hat{\gamma}_{x1}^{(i)} x_1 + \hat{\gamma}_{x2}^{(i)} x_2 + \dots + \hat{\gamma}_{x(i-1)}^{(i)} x_{i-1} + e_{xi} \,, \qquad \text{(Eq.2)}$$

for $2 \le i \le k$. Thus,

$$e_{x2} = -\hat{\gamma}_0^{(2)} - \hat{\gamma}_{x1}^{(2)} x_1 + x_2$$

$$e_{x3} = -\hat{\gamma}_0^{(3)} - \hat{\gamma}_{x1}^{(3)} x_1 - \hat{\gamma}_{x2}^{(3)} x_2 + x_3$$

$$\vdots$$

$$e_{xk} = -\hat{\gamma}_0^{(k)} - \hat{\gamma}_{x1}^{(k)} x_1 - \hat{\gamma}_{x2}^{(k)} x_2 - \dots - \hat{\gamma}_{x(k-1)}^{(k)} x_{k-1} + x_k \,. \qquad \text{(Eq.3)}$$

By its formulation, $e_{xi}$ represents the difference between the actual value of $x_i$ and the value of $x_i$ as predicted by all previous measurements of $x$.

### 1.1.3. Definition 3: Unexplained residuals (UR) models

We also define the UR model $\hat{y}_{UR}^{(i)}$ – an OLS regression model which represents $y$ as a function of $1, x_1, e_{x2}, \dots, e_{xi}$, for $1 \le i \le k$ – as:

$$\hat{y}_{UR}^{(1)} = \hat{\lambda}_0^{(1)} + \hat{\lambda}_{x1}^{(1)} x_1$$

$$\hat{y}_{UR}^{(2)} = \hat{\lambda}_0^{(2)} + \hat{\lambda}_{x1}^{(2)} x_1 + \hat{\lambda}_{ex2}^{(2)} e_{x2}$$

⋮

$$\hat{y}_{UR}^{(k)} = \hat{\lambda}_0^{(k)} + \hat{\lambda}_{x1}^{(k)} x_1 + \hat{\lambda}_{ex2}^{(k)} e_{x2} + \cdots + \hat{\lambda}_{exk}^{(k)} e_{xk} \,. \qquad \text{(Eq.4)}$$

Thus, the final composite model $\hat{y}_{UR}^{(k)}$ represents the outcome as a function of the initial value of $x$ and all subsequent increases.

## 1.2. Mathematical proofs

The proofs that follow rely upon the following key properties of OLS regression estimators and require the following two lemmas:

*Key properties of OLS estimators:* We may represent the regression equation $y = \beta_0 + \beta_1 x_1 + \cdots + \beta_k x_k + \varepsilon$ in summary notation as:

$$y = X\beta + \varepsilon \,,$$

where: $y$ represents the vector of $n$ continuous observations of the outcome; $X$ represents the $n \times (k+1)$ matrix of $n$ observations for $k$ continuous covariates and 1 constant; $\beta$ represents the $k+1$ vector of coefficients for each covariate and constant; and $\varepsilon$ represents the vector of $n$ residuals.

The OLS estimate of $\beta$ is given by:

$$\hat{\beta} = (X'X)^{-1} X'y \,.$$

On the assumption that the inverse matrix exists, this equation has a unique solution.

Further, for the given OLS equation $y = X\hat{\beta} + e$, it can be shown that the vector of residuals ($e$) is orthogonal (denoted ⊥) to every column $(1, x_1, x_2, \ldots x_k)$ of $X$.

*Note that detailed proofs have not been provided, but can be located in referenced material [2].

*Lemma 1:* For two orthogonal components $\tau$ and $\delta$ (i.e. $\tau \perp \delta$), the estimated coefficients of the regression of $y$ on $\tau$ and $\delta$ are equal to the estimated coefficients for the separate regressions of $y$ on $\tau$ and $y$ on $\delta$.

*Proof of Lemma 1:* The regression of $y$ on $\tau$ and $\delta$ may be written as:

$$y = [\tau \quad \delta] \begin{bmatrix} \beta_\tau \\ \beta_\delta \end{bmatrix} + \epsilon = \tau\beta_\tau + \delta\beta_\delta + \varepsilon \,.$$

From Definition 1, the OLS estimate of $\beta_\tau$ and $\beta_\delta$ is given by $\hat{\beta} = (X'X)^{-1}X'y$. In this scenario,

$$X'X = \begin{bmatrix} \tau' \\ \delta' \end{bmatrix} [\tau \quad \delta] = \begin{bmatrix} \tau'\tau & \tau'\delta \\ \delta'\tau & \delta'\delta \end{bmatrix} = \begin{bmatrix} \tau'\tau & 0 \\ 0 & \delta'\delta \end{bmatrix},$$

where the final equivalency follows from the condition of orthogonality. Then

$$(X'X)^{-1} = \begin{bmatrix} \tau'\tau & 0 \\ 0 & \delta'\delta \end{bmatrix}^{-1} = \begin{bmatrix} (\tau'\tau)^{-1} & 0 \\ 0 & (\delta'\delta)^{-1} \end{bmatrix}$$

and

$$X'y = \begin{bmatrix} \tau' \\ \delta' \end{bmatrix} y = \begin{bmatrix} \tau' y \\ \delta' y \end{bmatrix}.$$

Combining these elements gives:

$$\begin{bmatrix}\hat{\beta}_\tau\\ \hat{\beta}_\delta\end{bmatrix}=\begin{bmatrix}(\tau'\tau)^{-1} & 0\\ 0 & (\delta'\delta)^{-1}\end{bmatrix}\begin{bmatrix}\tau' y\\ \delta' y\end{bmatrix}=\begin{bmatrix}(\tau'\tau)^{-1}\tau' y\\ (\delta'\delta)^{-1}\delta' y\end{bmatrix}.$$

From this, we see that the estimated coefficients are equivalent to those that would be produced for the separate regressions of $y$ on $\tau$ and $y$ on $\delta$. ■

***Lemma 2:*** If $\tau_i \perp \delta_j$ for $0 \le i \le h$ and $0 \le j \le k$, then $span(\tau_0, \tau_1, \dots, \tau_h) \perp span(\delta_0, \delta_1, \dots \delta_k)$ for any vectors $\tau_0, \tau_1, \dots, \tau_h, \delta_0, \delta_1, \dots \delta_k$.[1]

*Proof of Lemma 2:* $\tau_i \perp \delta_j$ implies that $\tau_i \cdot \delta_j = 0$ for $0 \le i \le h$ and $0 \le j \le k$. Then

$$\begin{aligned} & span(\tau_0, \tau_1, \dots, \tau_h) \cdot span(\delta_0, \delta_1, \delta_2, \dots \delta_k) \\ &\quad = (c_0\tau_0 + c_1\tau_1 + \cdots + c_h\tau_h) \cdot (d_0\delta_0 + d_1\delta_1 + \cdots + d_k\delta_k) \\ &\quad = c_0 d_0(\tau_0 \cdot \delta_0) + c_0 d_1(\tau_0 \cdot \delta_1) + \cdots + c_0 d_k(\tau_0 \cdot \delta_k) + c_1 d_0(\tau_1 \cdot \delta_0) + \\ &\qquad c_1 d_1(\tau_1 \cdot \delta_1) + \cdots + c_1 d_k(\tau_1 \cdot \delta_k) + \cdots + c_h d_0(\tau_h \cdot \delta_0) + \\ &\qquad c_h d_1(\tau_h \cdot \delta_1) + \cdots + c_h d_k(\tau_h \cdot \delta_k) \\ &\quad = c_0 d_0(0) + c_0 d_1(0) + \cdots + c_0 d_k(0) + c_1 d_0(0) + c_1 d_1(0) + \cdots + \\ &\qquad c_1 d_k(0) + \cdots + c_h d_0(0) + c_h d_1(0) + \cdots + c_h d_k(0) \\ &\quad = 0\,. \end{aligned}$$

Thus, $span(\tau_0, \tau_1, \dots, \tau_h) \perp span(\delta_0, \delta_1, \delta_2, \dots \delta_k)$. ■

## 1.2.1. Covariate orthogonality

We prove that all UR terms $e_{x2}, e_{x3}, \dots, e_{xk}$ are orthogonal to all preceding variables in the composite UR model (Eq.3), and therefore orthogonal to their span; we prove this below.

***Lemma 3:*** $\boldsymbol{e_{xi}} \perp \boldsymbol{e_{x2}}, \boldsymbol{e_{x3}}, \dots, \boldsymbol{e_{x(i-1)}}$, for $2 \le i \le k$.

*Proof of Lemma 3:* By construction, $e_i$ represents the residuals from the OLS regression of $x_i \sim 1, x_1, x_2, \dots, x_{i-1}$. Thus, $e_{xi} \perp 1, x_1, x_2, \dots, x_{i-1}$, which implies that $e_{xi} \perp span(1, x_1, x_2, \dots, x_{i-1})$ by Lemma 2.

It is clear that $e_{x2}, e_{x3}, \dots, e_{x(i-1)} \in span(1, x_1, x_2, \dots, x_{i-1})$ for $2 \le i \le k$ by construction; we are therefore able to conclude that $e_{xi} \perp e_{x2}, e_{x3}, \dots, e_{x(i-1)}$. ■

***Theorem 1:*** $\boldsymbol{e_{xi}} \perp \boldsymbol{span(1, x_1, e_{x2}, e_{x3}, \dots, e_{x(i-1)})}$, for $2 \le i \le k$.

*Proof of Theorem 1:* $e_{xi} \perp 1, x_1$ because $e_{xi}$ represents the residuals from the OLS regression of $x_i \sim 1, x_1, x_2, \dots, x_{i-1}$. Further, $e_{xi} \perp e_{x2}, e_{x3}, \dots, e_{x(i-1)}$ for $2 \le i \le k$ by Lemma 3.

Thus, $e_{xi} \perp span(1, x_1, e_{x2}, e_{x3}, \dots, e_{x(i-1)})$ by Lemma 2. ■

---

[1] The span of a set of vectors $\delta_0, \delta_1, \delta_2, \dots \delta_k$ is the set of all possible linear combinations of $\delta_0, \delta_1, \delta_2, \dots \delta_k$, i.e.:

$$span(\delta_0, \delta_1, \delta_2, \dots \delta_k) = c_0\delta_0 + c_1\delta_1 + c_2\delta_2 + \cdots + c_k\delta_k\,,$$

where the coefficients $c_0, c_1, c_2, \dots, c_k$ are scalars.

### 1.2.2. Property (i): $\hat{\boldsymbol{y}}_{\boldsymbol{S}}^{(\boldsymbol{k})} = \hat{\boldsymbol{y}}_{\boldsymbol{UR}}^{(\boldsymbol{k})}$

*Proof of Property (i):* This equality follows from the fact that each UR model $\hat{y}_{UR}^{(i)}$ is a function of the same variables as the corresponding standard regression model $\hat{y}_{S}^{(i)}$.

By Definition 3, $\hat{y}_{UR}^{(i)} = f(1, x_1, e_{x2}, \dots, e_{xi})$, where $e_{xi} = f(1, x_1, x_2, \dots, x_i)$ by Definition 2. Thus, it also holds that

$$\hat{y}_{UR}^{(i)} = f(1, x_1, x_2, \dots, x_i)\ .$$

Moreover, by Definition 1,

$$\hat{y}_{S}^{(i)} = f(1, x_1, x_2, \dots, x_i)\ .$$

From this, it follows that $\hat{y}_{S}^{(i)} = \hat{y}_{UR}^{(i)}$ and, consequently, $\hat{y}_{S}^{(k)} = \hat{y}_{UR}^{(k)}$. ■

### 1.2.3. Property (ii): $\hat{\boldsymbol{\alpha}}_{\boldsymbol{x1}}^{(\boldsymbol{1})} = \hat{\boldsymbol{\lambda}}_{\boldsymbol{x1}}^{(\boldsymbol{k})}$

*Proof of Property (ii):* By definition, $\hat{y}_{S}^{(1)} = \hat{y}_{UR}^{(1)} = f(1, x_1)$, and so it is trivially true that $\hat{\alpha}_{x1}^{(1)} = \hat{\lambda}_{x1}^{(1)}$.

Because $e_{xi} \perp span(1, x_1, e_{x2}, e_{x3}, \dots, e_{x(i-1)})$ for $2 \le i \le k$ by Theorem 1, we are able to apply Lemma 1 and conclude that $\hat{\lambda}_{x1}^{(1)} = \hat{\lambda}_{x1}^{(2)} = \cdots = \hat{\lambda}_{x1}^{(k)}$.

Therefore, $\hat{\alpha}_{x1}^{(1)} = \hat{\lambda}_{x1}^{(k)}$. ■ [2]

### 1.2.4. Property (iii): $\hat{\boldsymbol{\alpha}}_{\boldsymbol{xi}}^{(\boldsymbol{i})} = \hat{\boldsymbol{\lambda}}_{\boldsymbol{exi}}^{(\boldsymbol{k})}$

*Proof of Property (iii):* Consider the UR model:

$$\hat{y}_{UR}^{(i)} = \hat{\lambda}_{0}^{(i)} + \hat{\lambda}_{x1}^{(i)} x_1 + \hat{\lambda}_{ex2}^{(i)} e_{x2} + \cdots + \hat{\boldsymbol{\lambda}}_{\boldsymbol{exi}}^{(\boldsymbol{i})} e_{xi}\ .$$

If we substitute the expansion for $e_{xi}$ (Eq.3) into this equation and rearrange, we produce:

$$\begin{aligned}\hat{y}_{UR}^{(i)} &= \hat{\lambda}_{0}^{(i)} + \hat{\lambda}_{x1}^{(i)} x_1 + \hat{\lambda}_{ex2}^{(i)}[-\hat{\gamma}_{0}^{(2)} - \hat{\gamma}_{x1}^{(2)} x_1 + x_2] + \cdots + \hat{\lambda}_{exi}^{(i)}[-\hat{\gamma}_{0}^{(i)} - \hat{\gamma}_{x1}^{(i)} x_1 - \hat{\gamma}_{x2}^{(i)} x_2 - \\ &\quad \cdots - \hat{\gamma}_{x(i-1)}^{(i)} x_{i-1} + x_i] \\ &= [\hat{\lambda}_{0}^{(i)} - \hat{\lambda}_{ex2}^{(i)} \gamma_{0}^{(2)} - \cdots - \hat{\lambda}_{exi}^{(i)} \gamma_{0}^{(i)}] + \left[\hat{\lambda}_{x1}^{(i)} - \hat{\lambda}_{ex2}^{(i)} \gamma_{x1}^{(2)} - \cdots - \hat{\lambda}_{exi}^{(i)} \gamma_{x1}^{(i)}\right] x_1 + \\ &\quad \left[\hat{\lambda}_{ex2}^{(i)} - \hat{\lambda}_{ex3}^{(i)} \gamma_{x2}^{(3)} - \cdots - \hat{\lambda}_{exi}^{(i)} \gamma_{x2}^{(i)}\right] x_2 + \cdots + \left[\hat{\boldsymbol{\lambda}}_{\boldsymbol{exi}}^{(\boldsymbol{i})}\right] x_i\ .\end{aligned}$$

Since we have already established that $\hat{y}_{S}^{(i)} = \hat{y}_{UR}^{(i)}$ (i.e. Property (i)) because they are functions of the same covariates, it follows that the estimated coefficients for those covariates must themselves be equal. Specifically, we are able to see that the coefficient for $x_i$ will always equal the coefficient for $e_{xi}$, i.e. $\hat{\alpha}_{xi}^{(i)} = \hat{\lambda}_{exi}^{(i)}$.

Finally, because $e_{xi} \perp span(1, x_1, e_{x2}, e_{x3}, \dots, e_{x(i-1)})$, we can again apply Lemma 1 and conclude that $\hat{\lambda}_{exi}^{(1)} = \hat{\lambda}_{exi}^{(2)} = \cdots = \hat{\lambda}_{exi}^{(k)}$, from which it follows that $\hat{\alpha}_{xi}^{(i)} = \hat{\lambda}_{exi}^{(k)}$.■

---

[2] Although no causal meaning/significance can be attributed to the intercept term, the logic applied in this proof may be easily extended to show that $\hat{\alpha}_{0}^{(1)} = \hat{\lambda}_{0}^{(k)}$.

# 2. Appendix 2: UR models: Time-invariant confounder

Proofs of Properties (i) – (iii) for the scenario depicted in Figure 2a (i.e. $k$ longitudinally measured exposure variables $x_1, x_2, \ldots, x_k$, one time-invariant confounder $m$, and one distal outcome $y$).

## 2.1. Definitions

We extend the definitions (1-3) provided in Appendix 1 to examine the scenario depicted in Figure 2a.

### 2.1.1. Definition 4: Standard regression models

Because the relationship between each measurement $x_i$ and $y$ is confounded by $m$ (for $1 \leq i \leq k$), adjustment for $m$ is necessary to estimate the total effect of $x_i$ and $y$ in the standard regression models:

$$\hat{y}_S^{(1)} = \hat{\alpha}_0^{(1)} + \hat{\alpha}_m^{(1)} m + \boldsymbol{\hat{\alpha}_{x1}^{(1)} x_1}$$

$$\hat{y}_S^{(2)} = \hat{\alpha}_0^{(2)} + \hat{\alpha}_m^{(2)} m + \hat{\alpha}_{x1}^{(2)} x_1 + \boldsymbol{\hat{\alpha}_{x2}^{(2)} x_2}$$

$$\vdots$$

$$\hat{y}_S^{(k)} = \hat{\alpha}_0^{(k)} + \hat{\alpha}_m^{(k)} m + \hat{\alpha}_{x1}^{(k)} x_1 + \hat{\alpha}_{x2}^{(k)} x_2 + \cdots + \boldsymbol{\hat{\alpha}_{xk}^{(k)} x_k}\,. \qquad \text{(Eq.5)}$$

### 2.1.2. Definition 5: Unexplained residual (UR) terms

In Figure 2a, it is clear that $m$ confounds the relationship between $x_i$ and $x_1, x_2, \ldots, x_{i-1}$ for $2 \leq i \leq k$, and thus adjustment for $m$ is necessary when regressing $x_i \sim x_1, x_2, \ldots, x_{i-1}$ to generate each UR term $e_{xi}$, i.e.:

$$x_i = \hat{\gamma}_0^{(i)} + \hat{\gamma}_m^{(i)} m + \hat{\gamma}_{x1}^{(i)} x_1 + \hat{\gamma}_{x2}^{(i)} x_2 + \cdots + \hat{\gamma}_{x(i-1)}^{(i)} x_{i-1} + e_{xi} \qquad \text{(Eq.6)}$$

and

$$e_{xi} = -\hat{\gamma}_0^{(i)} - \hat{\gamma}_m^{(i)} m - \hat{\gamma}_{x1}^{(i)} x_1 - \hat{\gamma}_{x2}^{(i)} x_2 - \cdots - \hat{\gamma}_{x(i-1)}^{(i)} x_{i-1} + x_i\,. \qquad \text{(Eq.7)}$$

In this way, $e_{xi}$ represents the difference between the actual value of $x_i$ and the value of $x_i$ as predicted by all previous measurements $x_1, x_2, \ldots, x_{i-1}$, *adjusted for the confounding effect of $m$.*

### 2.1.3. Definition 6: Unexplained residuals (UR) models

Furthermore, $m$ confounds the relationship between $x_1$ and $y$, and so adjustment must be made in the composite UR model:

$$\hat{y}_{UR}^{(k)} = \hat{\lambda}_0^{(k)} + \hat{\lambda}_m^{(k)} m + \hat{\lambda}_{x1}^{(k)} x_1 + \hat{\lambda}_{ex2}^{(k)} e_{x2} + \cdots + \hat{\lambda}_{exk}^{(k)} e_{xk}\,. \qquad \text{(Eq.8)}$$

## 2.2. Mathematical proofs

The proofs that follow rely upon the following key properties of OLS regression estimators and require the following two lemmas:

*Key properties of OLS estimators:* We may represent the regression equation $y = \beta_0 + \beta_1 x_1 + \cdots + \beta_k x_k + \varepsilon$ in summary notation as:

$$y = X\beta + \varepsilon ,$$

where: $y$ represents the vector of $n$ continuous observations of the outcome; $X$ represents the $n \times (k+1)$ matrix of $n$ observations for $k$ continuous covariates and 1 constant; $\beta$ represents the $k+1$ vector of coefficients for each covariate and constant; and $\varepsilon$ represents the vector of $n$ residuals.

The OLS estimate of $\beta$ is given by:

$$\hat{\beta} = (X'X)^{-1}X'y .$$

On the assumption that the inverse matrix exists, this equation has a unique solution.

Further, for the given OLS equation $y = X\hat{\beta} + e$, it can be shown that the vector of residuals ($e$) is orthogonal (denoted $\perp$) to every column $(1, x_1, x_2, \ldots x_k)$ of $X$.

*Note that detailed proofs have not been provided, but can be located in referenced material [2].

*Lemma 1:* For two orthogonal components $\tau$ and $\delta$ (i.e. $\tau \perp \delta$), the estimated coefficients of the regression of $y$ on $\tau$ and $\delta$ are equal to the estimated coefficients for the separate regressions of $y$ on $\tau$ and $y$ on $\delta$.

*Proof of Lemma 1:* The regression of $y$ on $\tau$ and $\delta$ may be written as:

$$y = [\tau \quad \delta] \begin{bmatrix} \beta_\tau \\ \beta_\delta \end{bmatrix} + \epsilon = \tau\beta_\tau + \delta\beta_\delta + \varepsilon .$$

From Definition 1, the OLS estimate of $\beta_\tau$ and $\beta_\delta$ is given by $\hat{\beta} = (X'X)^{-1}X'y$. In this scenario,

$$X'X = \begin{bmatrix} \tau' \\ \delta' \end{bmatrix} [\tau \quad \delta] = \begin{bmatrix} \tau'\tau & \tau'\delta \\ \delta'\tau & \delta'\delta \end{bmatrix} = \begin{bmatrix} \tau'\tau & 0 \\ 0 & \delta'\delta \end{bmatrix},$$

where the final equivalency follows from the condition of orthogonality. Then

$$(X'X)^{-1} = \begin{bmatrix} \tau'\tau & 0 \\ 0 & \delta'\delta \end{bmatrix}^{-1} = \begin{bmatrix} (\tau'\tau)^{-1} & 0 \\ 0 & (\delta'\delta)^{-1} \end{bmatrix}$$

and

$$X'y = \begin{bmatrix} \tau' \\ \delta' \end{bmatrix} y = \begin{bmatrix} \tau'y \\ \delta'y \end{bmatrix}.$$

Combining these elements gives:

$$\begin{bmatrix} \hat{\beta}_\tau \\ \hat{\beta}_\delta \end{bmatrix} = \begin{bmatrix} (\tau'\tau)^{-1} & 0 \\ 0 & (\delta'\delta)^{-1} \end{bmatrix} \begin{bmatrix} \tau'y \\ \delta'y \end{bmatrix} = \begin{bmatrix} (\tau'\tau)^{-1}\tau'y \\ (\delta'\delta)^{-1}\delta'y \end{bmatrix}.$$

From this, we see that the estimated coefficients are equivalent to those that would be produced for the separate regressions of $y$ on $\tau$ and $y$ on $\delta$. ■

*Lemma 2:* If $\tau_i \perp \delta_j$ for $0 \leq i \leq h$ and $0 \leq j \leq k$, then $span(\tau_0, \tau_1, \dots, \tau_h) \perp span(\delta_0, \delta_1, \dots \delta_k)$ for any vectors $\tau_0, \tau_1, \dots, \tau_h, \delta_0, \delta_1, \dots \delta_k$.[3]

*Proof of Lemma 2:* $\tau_i \perp \delta_j$ implies that $\tau_i \cdot \delta_j = 0$ for $0 \leq i \leq h$ and $0 \leq j \leq k$. Then

$$
\begin{aligned}
&span(\tau_0, \tau_1, \dots, \tau_h) \cdot span(\delta_0, \delta_1, \delta_2, \dots \delta_k) \\
&\quad = (c_0\tau_0 + c_1\tau_1 + \cdots + c_h\tau_h) \cdot (d_0\delta_0 + d_1\delta_1 + \cdots + d_k\delta_k) \\
&\quad = c_0d_0(\tau_0 \cdot \delta_0) + c_0d_1(\tau_0 \cdot \delta_1) + \cdots + c_0d_k(\tau_0 \cdot \delta_k) + c_1d_0(\tau_1 \cdot \delta_0) + \\
&\qquad c_1d_1(\tau_1 \cdot \delta_1) + \cdots + c_1d_k(\tau_1 \cdot \delta_k) + \cdots + c_hd_0(\tau_h \cdot \delta_0) + \\
&\qquad c_hd_1(\tau_h \cdot \delta_1) + \cdots + c_hd_k(\tau_h \cdot \delta_k) \\
&\quad = c_0d_0(0) + c_0d_1(0) + \cdots + c_0d_k(0) + c_1d_0(0) + c_1d_1(0) + \cdots + \\
&\qquad c_1d_k(0) + \cdots + c_hd_0(0) + c_hd_1(0) + \cdots + c_hd_k(0) \\
&\quad = 0
\end{aligned}
$$

Thus, $span(\tau_0, \tau_1, \dots, \tau_h) \perp span(\delta_0, \delta_1, \delta_2, \dots \delta_k)$. ■

## 2.2.1. Covariate orthogonality

We prove that all UR terms $e_{x2}, e_{x3}, \dots, e_{xk}$ are orthogonal to all preceding variables in the composite UR model (Eq.8), and therefore orthogonal to their span; we prove this below.

*Lemma 4:* $\boldsymbol{e_{xi}} \perp \boldsymbol{e_{x2}}, \boldsymbol{e_{x3}}, \dots, \boldsymbol{e_{x(i-1)}}$, for $2 \leq i \leq k$.

*Proof of Lemma 4:* By construction, $e_{xi}$ represents the residuals from the OLS regression of $x_i \sim 1, m, x_1, x_2, \dots, x_{i-1}$ (Eq.7). Thus, $e_{xi} \perp 1, m, x_1, x_2, \dots, x_{i-1}$, from which it follows that $e_{xi} \perp span(1, m, x_1, x_2, \dots, x_{i-1})$ by Lemma 2.

Because $e_{x2}, e_{x3}, \dots, e_{x(i-1)} \in span(1, m, x_1, x_2, \dots, x_{i-1})$ for $2 \leq i \leq k$ by construction, we are able to conclude that $e_{xi} \perp e_{x2}, e_{x3}, \dots, e_{x(i-1)}$. ■

*Theorem 2:* $\boldsymbol{e_{xi}} \perp \boldsymbol{span(1, m, x_1, e_{x2}, e_{x3}, \dots, e_{x(i-1)})}$, for $2 \leq i \leq k$.

*Proof of Theorem 2:* $e_{xi} \perp 1, m, x_1$ because $e_{xi}$ represents the residuals from the OLS regression of $x_i \sim 1, m, x_1, x_2, \dots, x_{i-1}$. Further, $e_{xi} \perp e_{x2}, e_{x3}, \dots, e_{x(i-1)}$ for $2 \leq i \leq k$ by Lemma 4 above.

Thus, $e_{xi} \perp span(1, m, x_1, e_{x2}, e_{x3}, \dots, e_{x(i-1)})$ by Lemma 2. ■

## 2.2.2. Property (i): $\boldsymbol{\hat{y}_S^{(k)}} = \boldsymbol{\hat{y}_{UR}^{(k)}}$

*Proof of Property (i):* As before, this equality follows from the fact that $\hat{y}_{UR}^{(i)}$ is a function of the same variables as $\hat{y}_S^{(i)}$.

By Definition 6, $\hat{y}_{UR}^{(i)} = f(1, m, x_1, e_{x2}, \dots, e_{xi})$, where $e_i = f(1, m, x_1, x_2, \dots, x_i)$ by Definition 5. Thus, it also holds that

[3] The span of a set of vectors $\delta_0, \delta_1, \delta_2, \dots \delta_k$ is the set of all possible linear combinations of $\delta_0, \delta_1, \delta_2, \dots \delta_k$, i.e.:

$$span(\delta_0, \delta_1, \delta_2, \dots \delta_k) = c_0\delta_0 + c_1\delta_1 + c_2\delta_2 + \cdots + c_k\delta_k,$$

where the coefficients $c_0, c_1, c_2, \dots, c_k$ are scalars.

$$\hat{y}_{UR}^{(i)} = f(1, m, x_1, x_2, \dots, x_i) \, .$$

Moreover, by Definition 4,

$$\hat{y}_{S}^{(i)} = f(1, m, x_1, x_2, \dots, x_i) \, .$$

From this, it follows that $\hat{y}_S^{(i)} = \hat{y}_{UR}^{(i)}$ and, consequently, $\hat{y}_S^{(k)} = \hat{y}_{UR}^{(k)}$. ■

### 2.2.3. Property (ii): $\hat{\boldsymbol{\alpha}}_{\boldsymbol{x1}}^{(\mathbf{1})} = \hat{\boldsymbol{\lambda}}_{\boldsymbol{x1}}^{(\boldsymbol{k})}$

*Proof of Property (ii):* By definition, $\hat{y}_S^{(1)} = \hat{y}_{UR}^{(1)} = f(1, m, x_1)$, and it is trivially true that $\hat{\alpha}_{x1}^{(1)} = \hat{\lambda}_{x1}^{(1)}$. Because $e_{xi} \perp span(1, m, x_1, e_{x2}, e_{x3}, \dots, e_{x(i-1)})$ for $2 \leq i \leq k$ by Theorem 2, we conclude that $\hat{\lambda}_{x1}^{(1)} = \hat{\lambda}_{x1}^{(2)} = \dots = \hat{\lambda}_{x1}^{(k)}$ from Lemma 1.

Therefore, $\hat{\alpha}_{x1}^{(1)} = \hat{\lambda}_{x1}^{(k)}$. ■ [4]

### 2.2.4. Property (iii): $\hat{\boldsymbol{\alpha}}_{\boldsymbol{xi}}^{(\boldsymbol{i})} = \hat{\boldsymbol{\lambda}}_{\boldsymbol{exi}}^{(\boldsymbol{k})}$

*Proof of Property (iii):* Consider the UR model:

$$\hat{y}_{UR}^{(i)} = \hat{\lambda}_0^{(i)} + \hat{\lambda}_m^{(i)} m + \hat{\lambda}_{x1}^{(i)} x_1 + \hat{\lambda}_{ex2}^{(i)} e_{x2} + \dots + \hat{\boldsymbol{\lambda}}_{\boldsymbol{exi}}^{(\boldsymbol{i})} e_{xi} \, .$$

If we substitute the expansion for $e_{xi}$ (Eq.7) into this equation and rearrange, we produce:

$$\begin{aligned} \hat{y}_{UR}^{(i)} &= \hat{\lambda}_0^{(i)} + \hat{\lambda}_m^{(i)} m + \hat{\lambda}_{x1}^{(i)} x_1 + \hat{\lambda}_{ex2}^{(i)} \left[ -\hat{\gamma}_0^{(2)} - \hat{\gamma}_{x1}^{(2)} x_1 + x_2 - \hat{\gamma}_m^{(2)} m \right] + \dots + \\ &\quad \hat{\lambda}_{exi}^{(i)} \left[ -\hat{\gamma}_0^{(i)} - \hat{\gamma}_{x1}^{(i)} x_1 - \hat{\gamma}_{x2}^{(i)} x_2 - \dots - \hat{\gamma}_{x(i-1)}^{(i)} x_{i-1} + x_i - \hat{\gamma}_m^{(i)} m \right] \\ &= \left[ \hat{\lambda}_0^{(i)} - \hat{\lambda}_{ex2}^{(i)} \gamma_0^{(2)} - \dots - \hat{\lambda}_{exi}^{(i)} \gamma_0^{(i)} \right] + \left[ \hat{\lambda}_{x1}^{(i)} - \hat{\lambda}_{ex2}^{(i)} \gamma_{x1}^{(2)} - \dots - \hat{\lambda}_{exi}^{(i)} \gamma_{x1}^{(i)} \right] x_1 + \\ &\quad \left[ \hat{\lambda}_{ex2}^{(i)} - \hat{\lambda}_{ex3}^{(i)} \gamma_{x2}^{(3)} - \dots - \hat{\lambda}_{exi}^{(i)} \gamma_{x2}^{(i)} \right] x_2 + \dots + \left[ \hat{\boldsymbol{\lambda}}_{\boldsymbol{exi}}^{(\boldsymbol{i})} \right] x_i + \left[ \hat{\lambda}_m^{(i)} - \hat{\lambda}_{ex2}^{(i)} \gamma_m^{(2)} - \right. \\ &\quad \left. \hat{\lambda}_{exi}^{(i)} \gamma_m^{(i)} \right] m \, . \end{aligned}$$

We have already established that $\hat{y}_S^{(i)} = \hat{y}_{UR}^{(i)}$ (i.e. Property (i)) because they are functions of the same covariates, so it follows that the estimated coefficients for those covariates must themselves be equal. Specifically, we see that the coefficient for $x_i$ will always equal the coefficient for $e_{xi}$, i.e. $\hat{\alpha}_{xi}^{(i)} = \hat{\lambda}_{exi}^{(i)}$.

Because $e_{xi} \perp span(1, m, x_1, e_{x2}, e_{x3}, \dots, e_{x(i-1)})$, we may apply Lemma 1 and conclude that $\hat{\lambda}_{exi}^{(1)} = \hat{\lambda}_{exi}^{(2)} = \dots = \hat{\lambda}_{exi}^{(k)}$, from which it follows that $\hat{\alpha}_{xi}^{(i)} = \hat{\lambda}_{exi}^{(k)}$. ■

[4] Although no causal meaning/significance can be attributed to the coefficient of the confounder $m$, the logic applied in this proof may be easily extended to show that $\hat{\alpha}_m^{(1)} = \hat{\lambda}_m^{(k)}$.

# 3. Appendix 3: UR models: Time-varying confounder

Proofs of Properties (i) – (iii) for the scenario depicted in Figure 3a (i.e. $k$ longitudinally measured exposure variables $x_1, x_2, \dots, x_k$, one time-varying confounder $m_1, m_2, \dots, m_k$, and one distal outcome $y$).

## 3.1. Definitions

We extend the definitions (1-3) provided in Appendix 1 to examine the scenario depicted in Figure 3a.

### 3.1.1. Definition 7: Standard regression models

In this scenario, the relationship between each $x_i$ and $y$ is confounded by all previous measurements of the exposure $x_1, x_2, \dots, x_{i-1}$, as well as all previous and current measurements of the confounder $m_1, m_2, \dots, m_i$ (for $1 \le i \le k$). These covariates must all be included in the standard regression models to obtain an unbiased estimate of the total causal effect of each measurement $x_i$ on $y$, i.e.:

$$\hat{y}_S^{(1)} = \hat{\alpha}_0^{(1)} + \hat{\alpha}_{m1}^{(1)} m_1 + \hat{\alpha}_{x1}^{(1)} x_1$$

$$\hat{y}_S^{(2)} = \hat{\alpha}_0^{(2)} + \hat{\alpha}_{m1}^{(2)} m_1 + \hat{\alpha}_{x1}^{(2)} x_1 + \hat{\alpha}_{m2}^{(2)} m_2 + \hat{\alpha}_{x2}^{(2)} x_2$$

$$\vdots$$

$$\hat{y}_S^{(k)} = \hat{\alpha}_0^{(k)} + \hat{\alpha}_{m1}^{(k)} m_1 + \hat{\alpha}_{x1}^{(k)} x_1 + \cdots + \hat{\alpha}_{mk}^{(k)} m_k + \hat{\alpha}_{xk}^{(k)} x_k \,. \qquad \text{(Eq.9)}$$

### 3.1.2. Definition 8: Unexplained residual (UR) terms

The DAG in Figure 3a also makes evident that the relationship between each measurement $x_i$ and all previous measurements of the exposure $x_1, x_2, \dots, x_{i-1}$ is confounded by all previous and current measurements of the confounder $m_1, m_2, \dots, m_i$, for $2 \le i \le k$. Thus, we create UR terms $e_{xi}$ for each measurement of the exposure variable $x_i$ by adjusting for $m_1, m_2, \dots, m_i$, i.e.:

$$x_i = \hat{\gamma}_0^{(i)} + \hat{\gamma}_{m1}^{(i)} m_1 + \hat{\gamma}_{x1}^{(i)} x_1 + \cdots + \hat{\gamma}_{m(i-1)}^{(i)} m_{i-1} + \hat{\gamma}_{x(i-1)}^{(i)} x_{i-1} + \hat{\gamma}_{mi}^{(i)} m_i + e_{xi} \qquad \text{(Eq.10)}$$

and

$$e_{xi} = -\hat{\gamma}_0^{(i)} - \hat{\gamma}_{m1}^{(i)} m_1 - \hat{\gamma}_{x1}^{(i)} x_1 - \cdots - \hat{\gamma}_{m(i-1)}^{(i)} m_{i-1} - \hat{\gamma}_{x(i-1)}^{(i)} x_{i-1} - \hat{\gamma}_{mi}^{(i)} m_i + x_i \,. \text{(Eq.11)}$$

In this way, $e_{xi}$ represents the difference between the observed value of $x_i$ and the value of $x_i$ as predicted by all previous measurements $x_1, x_2, \dots, x_{i-1}$, *adjusted for the confounding effects of* $m_1, m_2, \dots, m_i$.

Previous proofs have relied upon the orthogonality of the terms in the composite UR model (i.e. Theorems 1 and 2 in Appendices 1 and 2, respectively). This necessitates the creation of UR terms $e_{mi}$ for each measurement of the time-varying confounding variable $m_i$, for $2 \le i \le k$. Each $e_{mi}$ is derived from the OLS regression of $m_i$ on all previous values of the confounder $m_1, m_2, \dots, m_{i-1}$ and all previous values of the exposure $x_1, x_2, \dots, x_{i-1}$, i.e.:

$$m_i = \hat{\eta}_0^{(i)} + \hat{\eta}_{m1}^{(i)} m_1 + \hat{\eta}_{x1}^{(i)} x_1 + \cdots + \hat{\eta}_{m(i-1)}^{(i)} m_{i-1} + \hat{\eta}_{x(i-1)}^{(i)} x_{i-1} + e_{mi} \qquad \text{(Eq.12)}$$

and

$$e_{mi} = -\hat{\eta}_0^{(i)} - \hat{\eta}_{m1}^{(i)} m_1 - \hat{\eta}_{x1}^{(i)} x_1 - \cdots - \hat{\eta}_{m(i-1)}^{(i)} m_{i-1} - \hat{\eta}_{i-1}^{(i)} x_{i-1} + m_i \,. \qquad \text{(Eq.13)}$$

These adjustments follow from the DAG in Figure 3a, in which it is evident that $x_1, x_2, \dots, x_{i-1}$ confound the relationship between $m_i$ and $m_1, m_2, \dots, m_{i-1}$. Thus, $e_{mi}$ has a similar interpretation to the original UR terms, in that it represents the part of $m_i$ unexplained by all previous values $m_1, m_2, \dots, m_{i-1}$, *adjusted for the confounding effects of* $x_1, x_2, \dots, x_{i-1}$.

### 3.1.3. Definition 9: Unexplained residuals (UR) models

Finally, we represent the composite UR model as a function of the initial value of the exposure $x_1$ and all subsequent URs for the exposure $e_{x2}, e_{x3}, \dots, e_{xi}$, and the initial value of the confounder $m_1$ and all subsequent URs for the confounder $e_{m2}, e_{m3}, \dots, e_{mi}$:

$$\hat{y}_{UR}^{(k)} = \hat{\lambda}_0^{(k)} + \hat{\lambda}_{m1}^{(k)} m_1 + \hat{\lambda}_{x1}^{(k)} x_1 + \hat{\lambda}_{em2}^{(k)} e_{m2} + \hat{\lambda}_{ex2}^{(k)} e_{x2} + \cdots + \hat{\lambda}_{emk}^{(k)} e_{mk} + \hat{\lambda}_{exk}^{(k)} e_{xk} \,. \qquad \text{(Eq.14)}$$

## 3.2. Mathematical proofs

The proofs that follow rely upon the following key properties of OLS regression estimators and require the following two lemmas:

*Key properties of OLS estimators:* We may represent the regression equation $y = \beta_0 + \beta_1 x_1 + \cdots + \beta_k x_k + \varepsilon$ in summary notation as:

$$y = X\beta + \varepsilon \,,$$

where: $y$ represents the vector of $n$ continuous observations of the outcome; $X$ represents the $n \times (k+1)$ matrix of $n$ observations for $k$ continuous covariates and 1 constant; $\beta$ represents the $k+1$ vector of coefficients for each covariate and constant; and $\varepsilon$ represents the vector of $n$ residuals.

The OLS estimate of $\beta$ is given by:

$$\hat{\beta} = (X'X)^{-1} X'y \,.$$

On the assumption that the inverse matrix exists, this equation has a unique solution.

Further, for the given OLS equation $y = X\hat{\beta} + e$, it can be shown that the vector of residuals ($e$) is orthogonal (denoted $\perp$) to every column ($1, x_1, x_2, \dots x_k$) of $X$.

*Note that detailed proofs have not been provided, but can be located in referenced material [2].

*Lemma 1:* For two orthogonal components $\tau$ and $\delta$ (i.e. $\tau \perp \delta$), the estimated coefficients of the regression of $y$ on $\tau$ and $\delta$ are equal to the estimated coefficients for the separate regressions of $y$ on $\tau$ and $y$ on $\delta$.

*Proof of Lemma 1:* The regression of $y$ on $\tau$ and $\delta$ may be written as:

$$y = [\tau \quad \delta] \begin{bmatrix} \beta_\tau \\ \beta_\delta \end{bmatrix} + \epsilon = \tau\beta_\tau + \delta\beta_\delta + \varepsilon \,.$$

From Definition 1, the OLS estimate of $\beta_\tau$ and $\beta_\delta$ is given by $\hat{\beta} = (X'X)^{-1} X'y$. In this scenario,

$$X'X = \begin{bmatrix} \tau' \\ \delta' \end{bmatrix} [\tau \quad \delta] = \begin{bmatrix} \tau'\tau & \tau'\delta \\ \delta'\tau & \delta'\delta \end{bmatrix} = \begin{bmatrix} \tau'\tau & 0 \\ 0 & \delta'\delta \end{bmatrix},$$

where the final equivalency follows from the condition of orthogonality. Then

$$(X'X)^{-1} = \begin{bmatrix} \tau'\tau & 0 \\ 0 & \delta'\delta \end{bmatrix}^{-1} = \begin{bmatrix} (\tau'\tau)^{-1} & 0 \\ 0 & (\delta'\delta)^{-1} \end{bmatrix}$$

and

$$X'y = \begin{bmatrix} \tau' \\ \delta' \end{bmatrix} y = \begin{bmatrix} \tau' y \\ \delta' y \end{bmatrix}.$$

Combining these elements gives:

$$\begin{bmatrix} \hat{\beta}_\tau \\ \hat{\beta}_\delta \end{bmatrix} = \begin{bmatrix} (\tau'\tau)^{-1} & 0 \\ 0 & (\delta'\delta)^{-1} \end{bmatrix} \begin{bmatrix} \tau' y \\ \delta' y \end{bmatrix} = \begin{bmatrix} (\tau'\tau)^{-1}\tau' y \\ (\delta'\delta)^{-1}\delta' y \end{bmatrix}.$$

From this, we see that the estimated coefficients are equivalent to those that would be produced for the separate regressions of $y$ on $\tau$ and $y$ on $\delta$. ■

*Lemma 2:* If $\tau_i \perp \delta_j$ for $0 \le i \le h$ and $0 \le j \le k$, then $span(\tau_0, \tau_1, \dots, \tau_h) \perp span(\delta_0, \delta_1, \dots \delta_k)$ for any vectors $\tau_0, \tau_1, \dots, \tau_h, \delta_0, \delta_1, \dots \delta_k$.[5]

*Proof of Lemma 2:* $\tau_i \perp \delta_j$ implies that $\tau_i \cdot \delta_j = 0$ for $0 \le i \le h$ and $0 \le j \le k$. Then

$$\begin{aligned} &span(\tau_0, \tau_1, \dots, \tau_h) \cdot span(\delta_0, \delta_1, \delta_2, \dots \delta_k) \\ &= (c_0\tau_0 + c_1\tau_1 + \cdots + c_h\tau_h) \cdot (d_0\delta_0 + d_1\delta_1 + \cdots + d_k\delta_k) \\ &= c_0d_0(\tau_0 \cdot \delta_0) + c_0d_1(\tau_0 \cdot \delta_1) + \cdots + c_0d_k(\tau_0 \cdot \delta_k) + c_1d_0(\tau_1 \cdot \delta_0) + \\ &\quad c_1d_1(\tau_1 \cdot \delta_1) + \cdots + c_1d_k(\tau_1 \cdot \delta_k) + \cdots + c_hd_0(\tau_h \cdot \delta_0) + \\ &\quad c_hd_1(\tau_h \cdot \delta_1) + \cdots + c_hd_k(\tau_h \cdot \delta_k) \\ &= c_0d_0(0) + c_0d_1(0) + \cdots + c_0d_k(0) + c_1d_0(0) + c_1d_1(0) + \cdots + \\ &\quad c_1d_k(0) + \cdots + c_hd_0(0) + c_hd_1(0) + \cdots + c_hd_k(0) \\ &= 0 \end{aligned}$$

Thus, $span(\tau_0, \tau_1, \dots, \tau_h) \perp span(\delta_0, \delta_1, \delta_2, \dots \delta_k)$. ■

### 3.2.1. Covariate orthogonality

Here, we show that: the UR terms for each measurement of the confounder (i.e. $e_{m2}, e_{m3}, \dots, e_{mi}$) are mutually orthogonal; the UR terms for each measurement of the exposure (i.e. $e_{x2}, e_{x3}, \dots, e_{xi}$) are mutually orthogonal; and, importantly, the UR terms $e_{m2}, e_{m3}, \dots, e_{mi}$ are orthogonal to $e_{x2}, e_{x3}, \dots, e_{xi}$.

*Lemma 6:* $\boldsymbol{e_{mi} \perp e_{m2}, e_{m3}, \dots, e_{m(i-1)}}$, for $2 \le i \le k$.

*Proof of Lemma 6:* By construction, $e_{mi}$ represents the residuals from the OLS regression of $m_i \sim 1, m_1, x_1, \dots, m_{i-1}, x_{i-1}$ (Eq.13). Thus, $e_{mi} \perp 1, m_1, x_1, \dots, m_{i-1}, x_{i-1}$, which implies $e_{mi} \cdot 1 = 0$, $e_{mi} \cdot m_1 = 0$, $e_{mi} \cdot x_1 = 0$, …, $e_{mi} \cdot m_{i-1} = 0$, $e_{mi} \cdot x_{i-1} = 0$.

From this, it follows that $e_{mi} \perp span(1, m_1, x_1, \dots, m_{i-1}, x_{i-1})$ from Lemma 2.

---

[5] The span of a set of vectors $\delta_0, \delta_1, \delta_2, \dots \delta_k$ is the set of all possible linear combinations of $\delta_0, \delta_1, \delta_2, \dots \delta_k$, i.e.:

$$span(\delta_0, \delta_1, \delta_2, \dots \delta_k) = c_0\delta_0 + c_1\delta_1 + c_2\delta_2 + \cdots + c_k\delta_k,$$

where the coefficients $c_0, c_1, c_2, \dots, c_k$ are scalars.

Because $e_{m2}, e_{m3}, \ldots, e_{m(i-1)} \epsilon\ span(1, m_1, x_1, \ldots, m_{i-1}, x_{i-1})$ for $2 \le i \le k$ by construction, we are able to conclude that $e_{mi} \perp e_{m2}, e_{m3}, \ldots, e_{m(i-1)}$. ■

<u>*Lemma 7:*</u> $\boldsymbol{e_{xi}} \perp \boldsymbol{e_{x2}}, \boldsymbol{e_{x3}}, \ldots, \boldsymbol{e_{x(i-1)}}$, for $2 \le i \le k$.

<u>*Proof of Lemma 7:*</u> By construction, $e_{xi}$ represents the residuals from the OLS regression of $x_i \sim 1, m_1, x_1, \ldots, m_{i-1}, x_{i-1}, m_i$ (Eq.12). Thus, $e_{xi} \perp 1, m_1, x_1, \ldots, m_{i-1}, x_{i-1}, m_i$, which implies $e_{xi} \cdot 1 = 0$, $e_{xi} \cdot m_1 = 0$, $e_{xi} \cdot x_1 = 0$, …, $e_{xi} \cdot m_{i-1} = 0$, $e_{xi} \cdot x_{i-1} = 0$, $e_{xi} \cdot m_i = 0$.

From this, it follows that $e_{xi} \perp span(1, m_1, x_1, \ldots, m_{i-1}, x_{i-1}, m_i)$ from Lemma 2.

Because $e_{x2}, e_{x3}, \ldots, e_{x(i-1)} \epsilon\ span(1, m_1, x_1, \ldots, m_{i-1}, x_{i-1}, m_i)$ for $2 \le i \le k$ by construction, we are able to conclude that $e_{xi} \perp e_{x2}, e_{x3}, \ldots, e_{x(i-1)}$. ■

<u>*Lemma 8:*</u> $\boldsymbol{e_{xi}} \perp \boldsymbol{e_{mj}}$, for $2 \le i \le k$ and $2 \le j \le k$.

<u>*Proof of Lemma 8:*</u> As established previously, $e_{xi} \perp span(1, m_1, x_1, \ldots, m_{i-1}, x_{i-1}, m_i)$ by Lemma 2, for $2 \le i \le k$. Because $e_{m2}, e_{m3}, \ldots, e_{mi} \epsilon\ span(1, m_1, x_1, \ldots, m_{i-1}, x_{i-1}, m_i)$ by construction, it is evident that $e_{xi} \perp e_{m2}, e_{m3}, \ldots, e_{mi}$.

Further, $e_{mj} \perp span(1, m_1, x_1, \ldots, m_{j-1}, x_{j-1})$ by Lemma 2, for $2 \le j \le k$. Because $e_{x2}, e_{x3}, \ldots, e_{x(j-1)} \epsilon\ span(1, m_1, x_1, \ldots, m_{j-1}, x_{j-1})$ by construction, it is evident that $e_{mj} \perp e_{x2}, e_{x3}, \ldots, e_{x(j-1)}$.

Combining these two results, it follows that $e_{xi} \perp e_{mj}$ for $2 \le i \le k$ and $2 \le j \le k$. ■

<u>*Theorem 3:*</u> $\boldsymbol{span(e_{xi}, e_{mi}) \perp span(1, m_1, x_1, \ldots, e_{m(i-1)}, e_{x(i-1)})}$, for $2 \le i \le k$.

<u>*Proof of Theorem 3:*</u> By definition, $e_{xi} \perp 1, m_1, x_1$. As established in Lemmas 7 and 8, $e_{xi} \perp e_{x2}, \ldots, e_{x(i-1)}, e_{m1}, \ldots, e_{m(i-1)}$.

Further, $e_{mi} \perp 1, m_1, x_1$ by definition, and as established in Lemmas 6 and 8, $e_{mi} \perp e_{x2}, \ldots, e_{x(i-1)}, e_{m1}, \ldots, e_{m(i-1)}$.

Thus, by Lemma 2, it follows that $span(e_{xi}, e_{mi}) \perp span(1, m_1, x_1, \ldots, e_{m(i-1)}, e_{x(i-1)})$. ■

## 3.2.2. Property (i): $\boldsymbol{\hat{y}_S^{(k)} = \hat{y}_{UR}^{(k)}}$

<u>*Proof of Property (i):*</u> As previously, Property (i) follows from the fact that $\hat{y}_{UR}^{(i)}$ is a function of the same variables as $\hat{y}_S^{(i)}$.

By Definition 9, $\hat{y}_{UR}^{(i)} = f(1, m_1, x_1, e_{m2}, e_{x2}, \ldots, e_{mi}, e_{xi})$, where $e_{xi} = f(1, m_1, x_1, \ldots, m_i, x_i)$ and $e_{mi} = f(1, m_1, x_1, \ldots, m_{i-1}, x_{i-1}, m_i)$ by Definition 8. Thus, it also holds that

$$\hat{y}_{UR}^{(i)} = f(1, m_1, x_1, \ldots, m_i, x_i)\ .$$

Moreover, by Definition 7,

$$\hat{y}_S^{(k)} = f(1, m_1, x_1, \ldots, m_i, x_i)\ .$$

From this, it follows that $\hat{y}_S^{(i)} = \hat{y}_{UR}^{(i)}$ and, consequently, $\hat{y}_S^{(k)} = \hat{y}_{UR}^{(k)}$. ■

### 3.2.3. Property (ii): $\hat{\boldsymbol{\alpha}}_{\boldsymbol{x1}}^{(\mathbf{1})} = \hat{\boldsymbol{\lambda}}_{\boldsymbol{x1}}^{(\boldsymbol{k})}$

_Proof of Property (ii):_ By definition, $\hat{y}_S^{(1)} = \hat{y}_{UR}^{(1)} = f(1, m_1, x_1)$, and it is trivially true that $\hat{\alpha}_{x1}^{(1)} = \hat{\lambda}_{x1}^{(1)}$.

Because $span(e_{xi}, e_{mi}) \perp span(1, m_1, x_1, \dots, e_{m(i-1)}, e_{x(i-1)})$ for $2 \le i \le k$ by Theorem 3, we are able to conclude that $\hat{\lambda}_{x1}^{(1)} = \hat{\lambda}_{x1}^{(2)} = \cdots = \hat{\lambda}_{x1}^{(k)}$ by applying Lemma 1.

Therefore, $\hat{\alpha}_{x1}^{(1)} = \hat{\lambda}_{x1}^{(k)}$. ■ [6]

### 3.2.4. Property (iii): $\hat{\boldsymbol{\alpha}}_{\boldsymbol{xi}}^{(\boldsymbol{i})} = \hat{\boldsymbol{\lambda}}_{\boldsymbol{exi}}^{(\boldsymbol{k})}$

_Proof of Property (iii):_ Consider the UR model:

$$\hat{y}_{UR}^{(i)} = \hat{\lambda}_0^{(i)} + \hat{\lambda}_{m1}^{(i)} m_1 + \hat{\lambda}_{x1}^{(i)} x_1 + \hat{\lambda}_{em2}^{(i)} e_{m2} + \hat{\lambda}_{ex2}^{(i)} e_{x2} + \cdots + \hat{\lambda}_{emi}^{(i)} e_{mi} + \hat{\boldsymbol{\lambda}}_{\boldsymbol{exi}}^{(\boldsymbol{i})} e_{xi} \,.$$

By substituting the expansions for $e_{xi}$ (Eq.11) and $e_{mi}$ (Eq.13) into this equation and rearranging, we produce:

$$\begin{aligned}\hat{y}_{UR}^{(i)} = {} & \hat{\lambda}_0^{(i)} + \hat{\lambda}_{m1}^{(i)} m_1 + \hat{\lambda}_{x1}^{(i)} x_1 + \hat{\lambda}_{em2}^{(i)} \left[-\hat{\eta}_0^{(2)} - \hat{\eta}_{x1}^{(2)} x_1 - \hat{\eta}_{m1}^{(2)} m_1 + m_2\right] + \\ & \hat{\lambda}_{ex2}^{(i)} \left[-\hat{\gamma}_0^{(2)} - \hat{\gamma}_{x1}^{(2)} x_1 + x_2 - \hat{\gamma}_{m1}^{(2)} m_1 - \hat{\gamma}_{m2}^{(2)} m_2\right] + \cdots + \hat{\lambda}_{emi}^{(i)} \left[-\hat{\eta}_0^{(i)} - \hat{\eta}_{x1}^{(i)} x_1 - \right. \\ & \left. \cdots - \hat{\eta}_{i-1}^{(i)} x_{i-1} - \hat{\eta}_{m1}^{(i)} m_1 - \cdots - \hat{\eta}_{m(i-1)}^{(i)} m_{i-1} + m_i\right] + \hat{\lambda}_{exi}^{(i)} \left[-\hat{\gamma}_0^{(i)} - \hat{\gamma}_{x1}^{(i)} x_1 - \cdots - \right. \\ & \left. \hat{\gamma}_{i-1}^{(i)} x_{i-1} + x_i - \hat{\gamma}_{m1}^{(i)} m_1 - \cdots - \hat{\gamma}_{mi}^{(i)} m_i\right] \\ = {} & \left[\hat{\lambda}_0^{(i)} - \hat{\lambda}_{em2}^{(i)} \hat{\eta}_0^{(2)} - \hat{\lambda}_{ex2}^{(i)} \hat{\gamma}_0^{(2)} - \cdots - \hat{\lambda}_{emi}^{(i)} \hat{\eta}_0^{(i)} - \hat{\lambda}_{exi}^{(i)} \hat{\gamma}_0^{(i)}\right] + \left[\hat{\lambda}_{m1}^{(i)} - \hat{\lambda}_{em2}^{(i)} \hat{\eta}_{m1}^{(2)} - \right. \\ & \left. \hat{\lambda}_{ex2}^{(i)} \hat{\gamma}_{m1}^{(2)} - \cdots - \hat{\lambda}_{emi}^{(i)} \hat{\eta}_{m1}^{(i)} - \hat{\lambda}_{exi}^{(i)} \hat{\gamma}_{m1}^{(i)}\right] m_1 + \left[\hat{\lambda}_{x1}^{(i)} - \hat{\lambda}_{em2}^{(i)} \hat{\eta}_{x1}^{(2)} - \hat{\lambda}_{ex2}^{(i)} \hat{\gamma}_{x1}^{(2)} - \cdots - \right. \\ & \left. \hat{\lambda}_{emi}^{(i)} \hat{\eta}_{x1}^{(i)} - \hat{\lambda}_{exi}^{(i)} \hat{\gamma}_{x1}^{(i)}\right] x_1 + \cdots + \left[\hat{\lambda}_{emi}^{(i)} - \hat{\lambda}_{exi}^{(i)} \hat{\gamma}_{mi}^{(i)}\right] m_i + \left[\hat{\boldsymbol{\lambda}}_{\boldsymbol{exi}}^{(\boldsymbol{i})}\right] x_i \,.\end{aligned}$$

Having established that $\hat{y}_S^{(i)} = \hat{y}_{UR}^{(i)}$ (i.e. Property (i)) because they are functions of the same covariates, it follows that the estimated coefficients for those covariates must themselves be equal. Specifically, we see that the coefficient for $x_i$ will always equal the coefficient for $e_{xi}$, i.e. $\hat{\alpha}_{xi}^{(i)} = \hat{\lambda}_{exi}^{(i)}$.

Finally, using the fact that $e_{xi} \perp span(1, m_1, x_1, e_{m2}, e_{x2}, \dots, e_{m(i-1)}, e_{x(i-1)}, e_{mi})$, we apply Lemma 1 and conclude that $\hat{\lambda}_{exi}^{(1)} = \hat{\lambda}_{exi}^{(2)} = \cdots = \hat{\lambda}_{exi}^{(k)}$, from which it follows that $\hat{\alpha}_{xi}^{(i)} = \hat{\lambda}_{exi}^{(k)}$. ■

[6] Although no causal meaning/significance can be attributed to the intercept term or the coefficients of the UR terms for the confounder $e_{m2}, \dots, e_{mk}$, the logic applied in this proof may be easily extended to show that $\hat{\alpha}_0^{(1)} = \hat{\lambda}_0^{(k)}$ and $\hat{\alpha}_{m2}^{(1)} = \hat{\lambda}_{em2}^{(k)}, \dots, \hat{\alpha}_{mk}^{(1)} = \hat{\lambda}_{emk}^{(k)}$, respectively.

# 4. Appendix 4: Details of standard error simulation

## 4.1. DAG

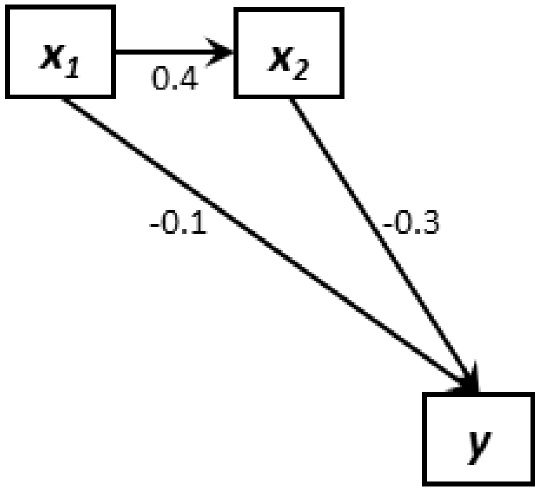


Path coefficients represent bivariate correlations.

## 4.2. Correlation matrix based upon DAG

| | $x_1$ | $x_2$ | $y$ |
|---|---|---|---|
| $x_1$ | 1.00 | - | - |
| $x_2$ | 0.40 | 1.00 | - |
| $y$ | -0.22 | -0.34 | 1.00 |

## 4.3. Population parameters used in simulation

| | *Mean* | *SD* |
|---|---|---|
| $x_1$ | 10.00 | 2.50 |
| $x_2$ | 15.00 | 3.75 |
| $y$ | 20.00 | 5.00 |

## 4.4. Annotated R code

```
# load packages required for simulation
require(Matrix); require(matrixcalc); require(MASS); require(dagitty); require(devtools)
# devtools::install_github("jtextor/dagitty/r") # update regularly

###################
## Covar FUNCTION ##
###################
```

```
# converts SDs and pairwise correlations to a covariace matrix

Covar <- function(n=2,SD=data.frame(1,1),c.vec=data.frame(0.5)) {
  check   <- n-length(SD)
  if (check !=0) stop("Incorrect SD specifications!")
  check   <- (n*(n-1)/2)-length(c.vec)
  if (check !=0) stop("Incorrect correlation specifications!")
  Cor     <- NULL
  for (i in 1:(n+1)) {
    Row <- NULL
    for (j in 1:(n+1)) {
      if (i==j) Element <- 1
      else if (i<j) Element <- c.vec[((i-1)*(2*n-i)/2)+(j-i)]
      else if (i>j) Element <- c.vec[((j-1)*(2*n-j)/2)+(i-j)]
      Row <- c(Row,Element)
    }
    Cor <- rbind(Cor,Row)
  } # cov(i,j) = cor(i,j)*sd(i)*sd(j)
  Cov <- matrix(nrow=n,ncol=n)
  for (i in 1:n) { for (j in 1:n) { Cov[i,j] <- Cor[i,j]*SD[i]*SD[j] }}
  Cov <- as.matrix(forceSymmetric(Cov))
  if (!is.positive.definite(Cov)) {
    print("Warning: covariance matrix made Positive Definite")
    Cov <- as.matrix(nearPD(Cov)$mat) }
  return(Cov)
}

#########
## DAG ##
#########

dag1 <- dagitty('dag{
		X1 [pos="0.2,0.2"]
		X2 [pos="0.6,0.2"]
		Y [pos="1,1"]
		X1 -> X2 [beta=0.4]
		X1 -> Y [beta=-0.1]
		X2 -> Y [beta=-0.3]
		}')
plot(dag1)
mod <- lm(Y~X1+X2, data=simulateSEM(dag1, empirical=TRUE))

#######################
## COVARIANCE MATRIX ##
#######################

MyData  <- simulateSEM(dag1, empirical=TRUE)  # standardised data
Names   <- c("X1","X2","Y")
SetCor  <- cor(MyData); Corr <- SetCor[lower.tri(SetCor)]
N       <- 1000
```

```
X1.mu   <- 10
X2.mu   <- 15
Y.mu    <- 20
Mu      <- c(X1.mu,X2.mu,Y.mu)
X1.sd   <- X1.mu/4
X2.sd   <- X2.mu/4
Y.sd    <- Y.mu/4
SD      <- c(X1.sd,X2.sd,Y.sd)
MyCov   <- Covar(3,SD,Corr)

################
## SIMULATION ##
################

# set storage for SEs for X1
seX1.reg <- NULL  # standard regression models
seX1.UR <- NULL  # UR models (as reported)
seX1.UR.boot <- NULL  # UR models (bootstrapped)

# set storage for SEs for X2/e2
seX2.reg <- NULL  # standard regression models
see2.UR <- NULL  # UR models (as reported)
see2.UR.boot <- NULL  # UR models (bootstrapped)

set.seed(23)

for (i in 1:1000) {
  # simulate N observations
  MyData  <- data.frame(mvrnorm(N,Mu,MyCov,empirical=FALSE)); names(MyData) <- Names

  # create standard regression model for X1 and save SE
  modX1 <- lm(Y~X1, data=MyData); seX1.reg <- c(seX1.reg, summary(modX1)$coefficients[2,2])

  # create standard regression model for X2 and save SE
  modX2 <- lm(Y~X1+X2, data=MyData); seX2.reg <- c(seX2.reg, summary(modX2)$coefficients[3,2])

  # create UR term
  modX2.resid <- lm(X2~X1, data=MyData); MyData$e2 <- modX2.resid$residuals

  # create UR model and save SEs for coeffs
  modUR <- lm(Y~X1+e2, data=MyData)
  seX1.UR <- c(seX1.UR, summary(modUR)$coefficients[2,2])
  see2.UR <- c(see2.UR, summary(modUR)$coefficients[3,2])

  # use bootstrapping to create distribution of coefficients for UR model
  coeffX1.UR.boot <- NULL  # set storage for coeffs for X1 from UR model
  coeffe2.UR.boot <- NULL  # set storage for coeffs for e2 from UR model

  for (j in 1:1000) {
    # select random sample with replacement from MyData
    select <- sample(c(1:1000), 1000, replace=TRUE)
```

```
    MyData.boot <- MyData[select,]

    # create UR term
    modX2.resid.boot <- lm(X2~X1, data=MyData.boot); MyData.boot$e2 <-
modX2.resid.boot$residuals

    # create UR models and save coeffs
    modUR.boot <- lm(Y~X1+e2, data=MyData.boot)
    coeffX1.UR.boot <- c(coeffX1.UR.boot, summary(modUR.boot)$coefficients[2,1])
    coeffe2.UR.boot <- c(coeffe2.UR.boot, summary(modUR.boot)$coefficients[3,1])
  }

  # calculate SES for UR model as standard deviation of distribution of coefficients
  seX1.UR.boot <- c(seX1.UR.boot, sd(coeffX1.UR.boot))
  see2.UR.boot <- c(see2.UR.boot, sd(coeffe2.UR.boot))
}

##################
## VIOLIN PLOTS ##
##################

# load required packages, import fonts
require(ggplot2); require(gridExtra); require(extrafont); require(Hmisc)
font_import(pattern="[C/c]alibri"); loadfonts(device="win") ## use fonttable() to see options

# function to produce summary statistics (mean and +/- sd)
data_summary <- function(x) {
  m <- mean(x)
  ymin <- m - sd(x)
  ymax <- m + sd(x)
  return(c(y=m, ymin=ymin, ymax=ymax))
}

# create stacked data frames for each pairwise comparison
DataFrameX1 <- stack(data.frame(seX1.reg,seX1.UR,seX1.UR.boot))
DataFrameX2 <- stack(data.frame(seX2.reg,see2.UR,see2.UR.boot))

# X1 plot
plotX1 <- ggplot(DataFrameX1, aes(x=ind, y=values)) +
  geom_violin(fill="gray60", color="gray30", size=1.2, trim=TRUE) +
  stat_summary(fun.data=data_summary, color="gray90", size=0.7) +
  scale_x_discrete(name="", labels=c("Standard \nregression \nmodels","Unexplained \nresiduals
\nmodels \n(reported)", "Unexplained \nresiduals \nmodels \n(bootstrapped)")) +
  scale_y_continuous(name="Standard error") +
  ggtitle("Exposure: x1") +
  theme_bw() +
  theme(axis.line=element_line(size=1, colour="black"),
        panel.border=element_blank(),
        #panel.grid.major=element_blank(),
        panel.grid.minor=element_blank(),
        plot.title=element_text(size=16, hjust = 0.5, family="Calibri"),
```

```
      text=element_text(size=13, family="Calibri Light"),
      axis.text.x=element_text(size=13),
      axis.text.y=element_text(size=11),
      plot.margin=unit(c(0.5,0.5,0.5,0.5),"cm"),
      legend.position="none")
#plotX1

# X2 plot
plotX2 <- ggplot(DataFrameX2, aes(x=ind, y=values)) +
  geom_violin(fill="gray60", color="gray30", size=1.2, trim=TRUE) +
  stat_summary(fun.data=data_summary, color="gray90", size=0.7) +
  scale_x_discrete(limits=c("seX2.reg", "see2.UR", "see2.UR.boot"), name="", labels=c("Standard
\nregression \nmodels","Unexplained \nresiduals \nmodels \n(reported)", "Unexplained \nresiduals
\nmodels \n(bootstrapped)")) +
  scale_y_continuous(name="Standard error") +
  ggtitle("Exposure: x2") +
  theme_bw() +
  theme(axis.line=element_line(size=1, colour="black"),
      panel.border=element_blank(),
      #panel.grid.major=element_blank(),
      panel.grid.minor=element_blank(),
      plot.title=element_text(size=16, hjust = 0.5, family="Calibri"),
      text=element_text(size=13, family="Calibri Light"),
      axis.text.x=element_text(size=13),
      axis.text.y=element_text(size=11),
      plot.margin=unit(c(0.5,0.5,0.5,0.5),"cm"),
      legend.position="none")
#plotX2

# composite plot
composite <- grid.arrange(plotX1, plotX2,
                    ncol=2, nrow=1,
                    widths=c(5,5), heights=8)
```

# References

1. Keijzer-Veen MG, Euser AM, Van Montfoort N, Dekker FW, Vandenbroucke JP and Van Houwelingen HC. A regression model with unexplained residuals was preferred in the analysis of the fetal origins of adult diseases hypothesis. *Journal of Clinical Epidemiology*. 2005; 58: 1320-4, DOI 10.1016/j.jclinepi.2005.04.004.
2. Gentle JE. *Matrix Algebra: Theory, Computations. and Applications in Statistics*. New York: Springer, 2007.
